# AGO2a but not AGO2b mediates antiviral defense against infection of wild-type cucumber mosaic virus in tomato

**DOI:** 10.1093/hr/uhad043

**Published:** 2023-03-13

**Authors:** Liling Zhao, Yingfang Chen, Xingming Xiao, Haiying Gao, Jiamin Cao, Zhongkai Zhang, Zhongxin Guo

**Affiliations:** Vector-borne Virus Research Center, State Key Laboratory for Ecological Pest Control of Fujian and Taiwan Crops, College of Plant Protection, Fujian Agriculture and Forestry University, Fuzhou 350002 China; Key Laboratory of Agricultural Biotechnology of Yunnan Province, Biotechnology and Germplasm Resources Research Institute, Yunnan Academy of Agricultural Sciences, Kunming, 650221 China; Vector-borne Virus Research Center, State Key Laboratory for Ecological Pest Control of Fujian and Taiwan Crops, College of Plant Protection, Fujian Agriculture and Forestry University, Fuzhou 350002 China; Vector-borne Virus Research Center, State Key Laboratory for Ecological Pest Control of Fujian and Taiwan Crops, College of Plant Protection, Fujian Agriculture and Forestry University, Fuzhou 350002 China; Vector-borne Virus Research Center, State Key Laboratory for Ecological Pest Control of Fujian and Taiwan Crops, College of Plant Protection, Fujian Agriculture and Forestry University, Fuzhou 350002 China; Vector-borne Virus Research Center, State Key Laboratory for Ecological Pest Control of Fujian and Taiwan Crops, College of Plant Protection, Fujian Agriculture and Forestry University, Fuzhou 350002 China; Key Laboratory of Agricultural Biotechnology of Yunnan Province, Biotechnology and Germplasm Resources Research Institute, Yunnan Academy of Agricultural Sciences, Kunming, 650221 China; Vector-borne Virus Research Center, State Key Laboratory for Ecological Pest Control of Fujian and Taiwan Crops, College of Plant Protection, Fujian Agriculture and Forestry University, Fuzhou 350002 China

## Abstract

Evolutionarily conserved antiviral RNA interference (RNAi) mediates a primary antiviral innate immunity preventing infection of broad-spectrum viruses in plants. However, the detailed mechanism in plants is still largely unknown, especially in important agricultural crops, including tomato. Varieties of pathogenic viruses evolve to possess viral suppressors of RNA silencing (VSRs) to suppress antiviral RNAi in the host. Due to the prevalence of VSRs, it is still unknown whether antiviral RNAi truly functions to prevent invasion by natural wild-type viruses in plants and animals. In this research, for the first time we applied CRISPR-Cas9 to generate *ago2a*, *ago2b*, or *ago2ab* mutants for two differentiated *Solanum lycopersicum* AGO2s, key effectors in antiviral RNAi. We found that AGO2a but not AGO2b was significantly induced to inhibit the propagation of not only VSR-deficient Cucumber mosaic virus (CMV) but also wild-type CMV-Fny in tomato; however, neither AGO2a nor AGO2b regulated disease induction after infection with either virus. Our findings firstly reveal a prominent role of AGO2a in antiviral RNAi innate immunity in tomato and demonstrate that antiviral RNAi evolves to defend against infection of natural wild-type CMV-Fny in tomato. However, AGO2a-mediated antiviral RNAi does not play major roles in promoting tolerance of tomato plants to CMV infection for maintaining health.

## Introduction

RNAi is an evolutionarily conserved mechanism in eukaryotes, and essentially regulates varieties of biological processes in organisms [1–3]. Antiviral RNAi is a fundamental antiviral innate immunity in plants and animals, playing vital roles in protecting hosts from infection of all kinds of viruses [4–6]. The core pathway of antiviral RNAi in plants has been proposed with the identification of several key components, mainly based on research in the model plant *Arabidopsis* [7–9]. In *Arabidopsis*, after viral infection the double-stranded viral RNA replication intermediate will be detected and processed into 21- to 24-bp duplex viral small inference RNAs (vsiRNAs) by different Dicer-like proteins (DCLs), which will produce primary vsiRNAs [10]. The primary duplex vsiRNAs will be loaded into effector Argonaute proteins (AGOs) to form the RNA-induced silencing complex (RISC) [11]. The passenger strand of duplex vsiRNA will be cleaved and then mature RISC will target complementary viral RNAs through the guide strand of vsiRNA, then AGOs in RISC will mediate the degradation or inhibit the translation of viral RNAs through post-translational gene silencing (PTGS) or transcriptional gene silencing (TGS) to restrict viral infection [12–14]. In the process, adequate secondary vsiRNA is produced through RNA-dependent polymerases (RDRs) by templating the erratic viral RNA to ensure efficient antiviral innate immunity [15–17]. A new class of virus-activated endogenous siRNA (vasiRNA) dependent on RDR1 was discovered in *Arabidopsis*, and may confer another layer of antiviral RNAi innate immunity in the plant [18, 19].

Notably, during the arms race between host and virus, viruses evolve to possess viral suppressors of RNA silencing (VSRs) to disturb antiviral RNAi at distinct steps in the pathway and to function as the key factor in viral virulence and pathogenesis [20–22]. The prevalence of VSRs and their potent inhibitory effect on antiviral RNAi have seriously hindered our appreciation of antiviral immunity. It is still questioned whether antiviral RNAi functions to counter infection of wild-type viruses in nature. On the other hand, the existence of VSRs also seriously hinders our effort to identify novel components in antiviral RNAi through genetic screening based on wild-type viruses. Most of the known components of antiviral RNAi, such as DCLs, AGOs, and RDRs, were identified based on their shared functions in silencing transgenes or endogenous genes [5, 23–25], and an applicable genetic screen for identifying specific components in antiviral RNAi was not available until an effective genetic screen to identify the *antiviral RNAi-defective* (avi) *Arabidopsis* mutant was developed recently through VSR 2b-deficient Cucumber mosaic virus (CMV-∆2b) [26–29]. The detailed mechanism of antiviral RNAi in plants is still unclear, especially in important agricultural crops.

Tomato is one of the most important agricultural crops in the world, valued at 102.6 billion US dollars in 2020, with yield estimated at 186.8 million tons in 2020 [30], and is often threatened by varieties of pathogenic plant viruses [31, 32]. It has been found that some known key components of the antiviral RNAi machinery, such as DCLs, AGOs, and RDRs, are conserved in the genome of tomato. However, their antiviral functions have not been systematically studied. Notably, tomato has evolved to possess multiple differentiated homologs for some of these key components, including AGO2s, of which AGO2a and AGO2b, two tandem repeated homologs, have evolved in tomato [33]. AGO2 is one of the key effectors forming RISC with 21- or 22-nucleotide siRNAs to specifically mediate antimicrobial defense in plants [16, 34]. AGO2 has been found to defend against infection with different species of viruses through antiviral RNAi in plants and animals [6]. In plants, it has been reported that AGO2 in *Arabidopsis* (AtAGO2) can limit infection with CMV, Turnip mosaic virus (TuMV), Potato virus X (PVX), and Turnip crinkle virus (TCV) in *Arabidopsis* [9, 16, 35–40], and an AGO2 homolog in *Nicotiana benthamiana* (NbAGO2) was also found to defend against infection with wild-type TuMV, PVX, TCV, Tomato ringspot virus (ToRSV), Tobacco mosaic virus (TMV), Sweet potato mild mottle virus (SPMMV), and Tomato bushy stunt virus (TBSV) in *N. benthamiana* [36, 41–45], though the AGO2 homolog in rice (OsAGO2) has been reported to increase plant susceptibility to Rice black-streaked dwarf virus [46]. Intriguingly, it was found that AtAGO2 or NbAGO2 can protect *Arabidopsis* or *N. benthamiana* from infection with CMV-∆2b but not wild-type CMV-Fny [16, 47]. However, the antiviral function of both AGO2a and AGO2b in tomato is still elusive and needs to be clarified with true knockout mutants [48].

CMV is an economically important plant pathogenic virus in the family *Bromoviridae*, infecting >1200 plant species, including important crops including tomatoes [49, 50]. It is also a model plant virus for studying the interaction between host plant and virus [51–53]. The CMV genome is composed of three single-stranded positive-sense RNAs that encode five viral proteins: helicase 1a protein, RNA-dependent RNA polymerase (RdRp) 2a protein, movement protein (MP), coat protein (CP), and VSR 2b protein [51, 54]. In previous research, we found that wild-type CMV-Fny can infect wild-type *Arabidopsis* plants or antiviral RNAi-defective *Arabidopsis* mutants and induce similar disease symptoms in these plants with comparable viral accumulation, due to the potent inhibitory effect of 2b on antiviral RNAi. However, CMV-∆2b can only abundantly accumulate and induce disease symptoms in antiviral RNAi-defective *Arabidopsis* mutants; it cannot efficiently infect and cause disease in wild-type *Arabidopsis* in which antiviral RNAi is intact [16, 26, 29]. Based on these findings, we established a robust platform to study antiviral RNAi in *Arabidopsis* through VSR-deficient CMV (CMV-Δ2b) [26, 29, 55]. Excitingly, we found that tomato is also a natural host of CMV. Therefore, CMV-∆2b together with wild-type CMV could also provide a powerful tool to dissect antiviral RNAi in tomato.

In this research we utilized CRISPR to generate *ago2a*, *ago2b* single-knockout, or *ago2ab* double-knockout mutants in our effort to dissect antiviral RNAi immunity in tomato with CMV-Fny and CMV-∆2b. It was found that *ago2a* but not *ago2b* displayed increased viral accumulation after infection of either CMV-Fny or CMV-∆2b, indicating that AGO2a but not AGO2b prevented infection of not only CMV-∆2b but also wild-type CMV in tomato. Surprisingly, *ago2a*, *ago2b*, or *ago2ab* did not show developmental defects or a difference in disease symptoms compared with wild-type tomato after infection with either virus, indicating they did not regulate plant development or disease symptom induction in tomato. We further found that AGO2a but not AGO2b was significantly induced after viral infection, and only AGO2a protein could be readily detected after transient expression, which may underlie their distinct function in antiviral immunity. Thus, in this research we developed an effective platform to study antiviral RNAi in tomato through CMV and its mutant variant, and our findings are not only the first to reveal a prominent role of AGO2a in antiviral RNAi innate immunity but also demonstrate that antiviral RNAi evolves to defend against infection of natural wild-type CMV in tomato.

## Results

### Generation of *ago2a*, *ago2b*, or *ago2ab* tomato mutants through CRISPR

AGO2a and AGO2b are respectively encoded by two tandem repeated homologs, Solyc02g069260 and Solyc02g069270, in tomato. Their full length is respectively composed of 1042 and 977 amino acids, and both contain five different domains: ArgoN, DUF1785, PAZ, ArgoL2, and Piwi from N-terminal to C-terminal (Supplementary Data Fig. S1a). AGO2a and AGO2b show high identity (80%) to each other (Supplementary Data Figs S1b andS2); both AGO2a and AGO2b also show high identity (>70%) to NbAGO2 (Supplementary Data Figs S1b andS2), although a specific 20-amino acid region in the ArgoN domain is missing in AGO2b compared with AGO2a or NbAGO2 or other AGO2 proteins (Supplementary Data Fig. S3). However, both AGO2a and AGO2b show high divergence from AtAGO2 (~80%) or OsAGO2 (~100%) (Supplementary Data Figs S1b andS2), and they lack the uncharacterized domain on the N-terminal of AtAGO2 or OsAGO2 (Supplementary Data Fig. S1a), suggesting that tomato AGO2a and AGO2b may function differently in antiviral RNAi compared with AtAGO2 or its homologs in other plants.

**Figure 1:**
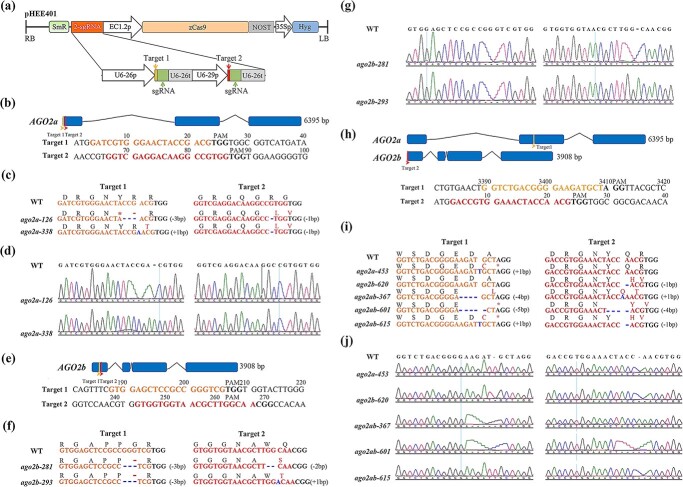
CRISPR/Cas9-mediated *AGO2a*, *AGO2b*, and *AGO2ab* mutations in Micro-Tom. **a** Schematic diagram to illustrate the construction of pHEE401 binary vector, used for editing the *AGO2a*, *AGO2b*, or *AGO2ab* gene in Micro-Tom. RB/LB, T-DNA right/left border; U6–26p and U6-29p, two *Arabidopsis* U6 gene promoters; U6–26t, U6–26 terminator with downstream sequence; EC1.2p, EC1.2 promoter; zCas9, *Zea mays* codon-optimized Cas9; Nost, *nos* gene terminator; 35Sp, CaMV 35S promoter; Hyg, hygromycin-resistance gene; SmR, streptomycin-resistance gene. In the structure of small guide RNA (sgRNA), the target (19 or 20 bp in length) is marked with yellow or red while the green part represents the 76-bp sgRNA scaffold. **b**, **e**, **h** Schematic illustration of two targets in the *AGO2a* gene (**b**), *AGO2b* gene (**e**), and *AGO2a/b* gene (**h**). Exons are indicated by rectangles and introns by lines. Positions of two sgRNA targets are indicated with orange and red arrowheads, respectively. Target sequences are highlighted with the same color as the target. PAM is indicated with bold font. **c**, **f**, **i** Mutations in *AGO2a* and *AGO2b* alleles of the *ago2a-126/338*/*453*, *ago2b-281*/*293/620*, and *ago2ab-367*/*601*/*615* transgenic lines. Sequences and encoded amino acids of sgRNA target regions are shown. Sequence changes by mutation are marked in blue and amino acid changes in red. **d**, **g**, **j** Sanger sequencing results of mutations. DNA fragments around the target sequences were amplified by PCR and then subjected to sequencing analysis.

We then utilized CRISPR-Cas9 to generate *ago2a*, *ago2b*, and *ago2ab* knockout mutants for the first time in order to characterize their function in antiviral RNAi in tomato, since AGO2a or AGO2b knockout mutants are still not available. For generating *ago2a* or *ago2b* single mutants, we designed two specific CRISPR guide RNAs targeting two different protospacer-adjacent motif (PAM) sites on the first exon of *AGO2a* (TGG^26–28^ and TGG^87–89^) (Fig. 1b) or the first exon of *AGO2b* (TGG^207–209^ and TGG^261–263^) (Fig. 1e). For generating the *ago2ab* double mutant, we designed two specific CRISPR guide RNAs which respectively targeted two different PAM sites on the second exon of *AGO2a* (AGG^3390–3409^) or the first exon of *AGO2b* (TGG^24–26^) (Fig. 1h). These CRISPR guide RNAs were respectively constructed into vector pHEE401, in which Cas9 was integrated, to produce three different expression vectors, pHEE401-AGO2a, pHEE401-AGO2b, and pHEE401-AGO2ab (Fig. 1a). They were further separately transformed into tomato Micro-Tom using the *Agrobacterium*-mediated method to edit target genes. We successfully obtained multiple transgenic lines for each transformation, and sequenced the targeting regions to identify homozygotic mutants, in which CAS9 was also segregated out, in *T*_2_ generation transgenic plants.

After confirmation by Sanger sequencing, we respectively obtained three allelic *ago2a*, *ago2b*, or *ago2ab* mutants. Among them, *ago2a-126* and *ago2a-338* respectively contain a 3- and 1-bp deletion, or a 1-bp insertion and 1-bp deletion in the target 1 region and target 2 region on *AGO2a* induced by pHEE401-AGO2a (Fig. 1c and d); *ago2a-453* only contains a 1-bp insertion in the target 1 region on *AGO2a* induced by pHEE401-AGO2ab (Fig. 1i and j). Mutant of *ago2b-281* or *ago2b-293* respectively contain a 3- and 2-bp deletion, or a 3-bp deletion and a 1-bp insertion in target 1 and target 2 on *AGO2b* induced by pHEE401-AGO2b (Fig. 1f and g), and *ago2b-620* only contains a 1-bp deletion in the target 2 region on *AGO2b* induced by pHEE401-AGO2ab (Fig. 1i and j). Double mutants of *ago2ab-367*, *ago2ab-601*, or *ago2ab-615* respectively contained a 4-bp deletion and a 1-bp insertion, a 5- and a 4-bp deletion, or a 1-bp insertion and a 1-bp deletion in target 1 on *AGO2a* and in target 2 on *AGO2b* induced by pHEE401-AGO2ab (Fig. 1i and j). All these small In-Del mutations were localized in exons of AGO2a or AGO2b and caused frameshift mutations in the *AGO2a* or *AGO2b* genes. Therefore, *ago2a*, *ago2b*, and *ago2ab* knockout mutants were successfully generated through CRISPR for further research.

### Antiviral immunity is compromised in tomato *ago2a* knockout mutants

**Figure 2:**
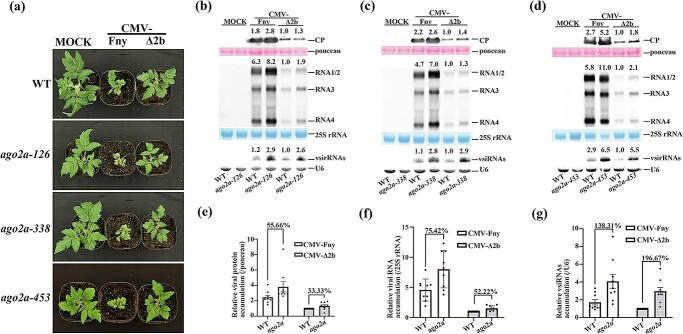
Antiviral immunity is compromised in tomato *ago2a* knockout mutants. **a** Wild-type (WT) and three lines of *ago2a* mutants (*126*, *338*, and *453*) were photographed 19 days post-inoculation (dpi) with buffer C (MOCK), CMV-Fny, or CMV-∆2b. Symptoms induced with CMV-Fny are shown in Supplementary Data Fig. S5. **b**–**d** Viral protein, viral RNA, and vsiRNA accumulation in WT and three lines of *ago2a* plants infected with CMV-Fny or CMV-∆2b at 19 dpi. Values at the top of the panels represent the corresponding hybridization signal intensity of viral CP, RNAs 1–4, and vsiRNAs. The intensity of WT inoculated with CMV-∆2b was set as 1. Ponceau, 25S rRNA, and U6 RNA were used as the loading controls. Blot experiments in each mutant line were repeated three times with similar results, shown in Supplementary Data Fig. S5. **e**–**g** Relative viral protein accumulation (**e**), viral RNA accumulation (**f**), and vsiRNA accumulation (**g**) in WT and *ago2a* plants infected with CMV-Fny or CMV-∆2b at 19 dpi. Values represent the average of nine individual blot-experiment results in three lines of *ago2a* with three technical replicates each. Error bars represent the standard deviation. The % sign indicates the percentage of increased accumulation in *ago2a* compared with WT (100%).

To find out the function of AGO2a in antiviral defense in tomato, we then respectively infected wild-type Micro-Tom and *ago2a-126*, *ago2a-338*, and *ago2a-453* mutants with wild-type CMV-Fny or CMV-Δ2b. We found that all mock *ago2a* mutants did not show developmental defects compared with wild-type Micro-Tom (Fig. 2a, Supplementary Data Fig.S4), indicating that AGO2a does not regulate plant growth and development. After CMV-Δ2b infection, these *ago2a* mutants did not show visible developmental defects either (Fig. 2a). However, 19 days after infection with wild-type CMV-Fny, both wild-type Micro-Tom and all three *ago2a* mutants developed severe disease symptoms, such as small stature, mosaic lesions, and curly leaves (Fig. 2a, Supplementary Data Fig.S5a). These results indicate that VSR 2b of CMV is critical to induce disease symptoms in tomato after viral infection. However, *ago2a* mutants did not show enhanced disease symptoms compared with wild-type tomato after infection with CMV-∆2b or CMV-Fny, indicating that AGO2a did not function importantly in preventing disease induction after viral infection in tomato.

To further find whether viral accumulation was affected in *ago2a* mutants, we examined viral CP protein and viral genomic RNAs in each of three allelic *ago2a* mutants by western blot or northern blot. It was found that the CP protein level was clearly increased in all three *ago2a* mutants after either CMV-Fny or CMV-Δ2b infection compared with wild-type Micro-Tom plants (Fig. 2b–d, top, Supplementary Data Fig. S5b–g, top). Consistently, viral genomic RNA accumulation was also dramatically increased in all three allelic *ago2a* mutants compared with wild-type Micro-Tom plants after infection with either wild-type CMV-Fny or CMV-Δ2b (Fig. 2b–d, middle, Supplementary Data Fig. S5b–g, middle). Northern blot analysis showed that the accumulation of viral siRNAs (vsiRNAs) in *ago2a* mutants was also increased compared with wild-type Micro-Tom plants (Fig. 2b–d, bottom, S5b–g, bottom), indicating that vsiRNA biogenesis was not affected in *ago2a* mutants.

To further demonstrate accumulation variation of CP, viral RNAs, and vsiRNAs in the *ago2a* mutant, we statistically calculated their relative accumulation based on three replicate results for each different *ago2a* mutant. It was found that, after infection CMV-Fny and CMV-Δ2b, viral CP was significantly increased by ~56 and ~33%, respectively, in the *ago2a* mutant compared with wild-type Micro-Tom (Fig. 2e), and viral RNA was significantly increased by ~75 and ~52% (Fig. 2f). Further RT–qPCR targeting viral *RdRp* also verified the significant elevation of viral accumulation in *ago2a* mutants (Supplementary Data Fig. S6a–c). But vsiRNAs was also significantly increased by ~138 and ~197% (Fig. 2g). These results indicate that AGO2a not only defends against infection with CMV-Δ2b but also functions to restrict infection with wild-type CMV virus in tomato. Surprisingly, this result is different from findings in *Arabidopsis*, in which wild-type CMV-Fny was efficiently propagated not only in wild-type *Arabidopsis* but also in antiviral RNAi-defective *Arabidopsis* plants because 2b potently inhibited antiviral RNAi in wild-type *Arabidopsis* [26, 56–58]. However, AGO2 does not directly regulate vsiRNA biogenesis in tomato, just like in *Arabidopsis* [16].

### AGO2b does not function in antiviral immunity in tomato

**Figure 3:**
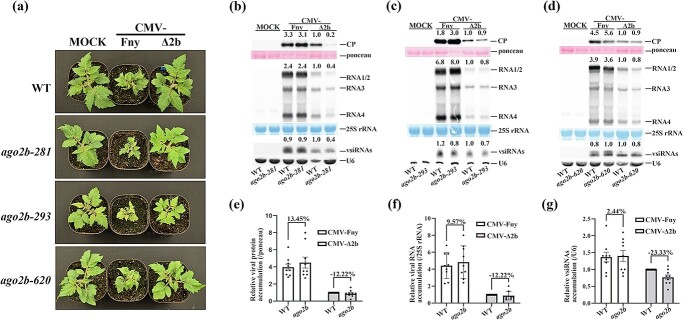
AGO2b does not function in antiviral immunity in tomato. **a** Wild-type (WT) and three lines of *ago2b* mutants (*281*, *293*, and *620*) were photographed at 19 dpi with buffer C (MOCK), CMV-Fny, or CMV-∆2b. Symptoms induced with CMV-Fny are shown in Supplementary Data Fig. S7. **b**–**d** Viral protein, viral RNA, and vsiRNA accumulation in WT and three lines of *ago2b* plants infected with CMV-Fny or CMV-∆2b at 19 dpi. Values at the top of the panels represent the corresponding hybridization signal intensity of viral CP, RNAs 1–4, and vsiRNAs. The intensity of WT inoculated with CMV-∆2b was set as 1. Ponceau, 25S rRNA, and U6 RNA were used as the loading controls. Blot experiments in each mutant line were repeated three times with similar results, shown in Supplementary Data Fig S7. **e**–**g** Relative viral protein accumulation (**e**), viral RNA accumulation (**f**), and vsiRNA accumulation (**g**) in WT and *ago2b* plants infected with CMV-Fny or CMV-∆2b at 19 dpi. Values represent the average of nine individual blot-experiment results in three lines of *ago2b* with three technical replicates each. Error bars represent the standard deviation. The % sign indicates the percentage of increased accumulation in *ago2b* compared with WT (100%).

To further investigate the antiviral function of AGO2b in tomato, we also infected *ago2b-281*, *ago2a-293*, and *ago2b-620* mutants along with wild-type Micro-Tom with wild-type CMV-Fny or CMV-∆2b, respectively. We found that all three mock *ago2b* mutants did not exhibit any visible defects in growth and development (Fig. 3a, Supplementary Data Fig.S4). After CMV-∆2b infection, none of three allelic *ago2b* mutants displayed observable disease symptoms compared with wild-type Micro-Tom (Fig. 3a). However, after infection with CMV-Fny, these *ago2b* mutants developed typical disease symptoms like wild-type Micro-Tom plants (Fig. 3a, Supplementary Data Fig.S7a). Thus, like AGO2a, AGO2b does not regulate plant growth and development in tomato either, and neither AGO2a nor AGO2b functions importantly to prevent disease symptom induction in tomato after viral infection.

We also further examined virus accumulation in these *ago2b* mutants compared with wild-type Micro-Tom. Unexpectedly, western or northern blot results respectively showed that, after infection with CMV-∆2b or CMV-Fny, the accumulation of CP protein, viral RNAs or vsiRNAs were not affected in all three different *ago2b* mutants compared with wild-type Micro-Tom (Fig. 3b–d, Supplementary Data Fig.S7b–g). To further demonstrate variation in accumulation of CP, viral RNAs, and vsiRNAs in the *ago2b* mutant, we also statistically calculated their relative accumulation based on three replicate results for each different *ago2b* mutant. It was found that, after infection with CMV-Fny or CMV-Δ2b, viral CP, RNAs, or vsiRNAs were not significantly changed in the *ago2b* mutant compared with wild-type Micro-Tom (Fig. 3e–g). Further RT–qPCR targeting viral *RdRp* showed a consistent result of viral accumulation in *ago2b* mutants (Supplementary Data Fig. S6d–f). These results indicate that, unlike AGO2a, AGO2b does not play major roles in antiviral RNAi in tomato.

### AGO2a and AGO2b do not function redundantly in antiviral defense and plant development in tomato

**Figure 4:**
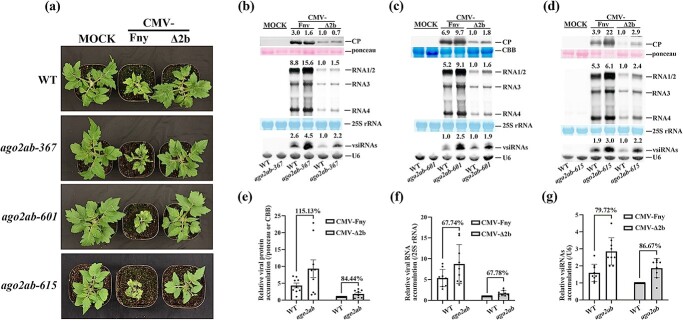
AGO2a and AGO2b do not function redundantly in antiviral defense and plant development in tomato. **a** Wild-type (WT) and three lines of *ago2ab* mutants (*367*, *601*, and *615*) were photographed at 19 dpi with buffer C (MOCK), CMV-Fny, or CMV-∆2b. Symptoms induced with CMV-Fny are shown in Supplementary Data Fig. S8a. **b**–**d** Viral protein, viral RNA, and vsiRNA accumulation in WT and three lines of *ago2ab* plants infected with CMV-Fny or CMV-∆2b at 19 dpi. Values at the top of the panels represent the corresponding hybridization signal intensity of viral CP, RNAs 1–4, and vsiRNAs. The intensity of WT inoculated with CMV-∆2b was set as 1. Ponceau, CBB, 25S rRNA, and U6 RNA were used as the loading controls. Blot experiments in each mutant line were repeated three times with similar results, shown in Supplementary Data Fig. S8. **e**–**g** Relative viral protein accumulation (**e**), viral RNA accumulation (**f**), and vsiRNA accumulation (**g**) in WT and *ago2ab* plants infected with CMV-Fny or CMV-∆2b at 19 dpi. Values represent the average of nine individual blot-experiment results for three lines of *ago2ab* with three technical replicates each. Error bars represent the standard deviation. The % sign indicates the percentage of increased accumulation in *ago2ab* compared with WT (100%).

We further found that tomato *ago2ab* double mutants did not exhibit any defects in growth and development compared with *ago2a*, *ago2b* single mutants or wild-type Micro-Tom plants (Fig. 4a, Supplementary Data Fig.S4), indicating that AGO2a and AGO2b do not play redundant roles in regulating plant development. To further find out whether AGO2a and AGO2b play redundant roles in antiviral defense, we infected *ago2ab-367*, *ago2ab-601*, or *ago2ab-615* double mutants along with wild-type Micro-Tom plants with wild-type CMV-Fny or CMV-∆2b, respectively. It was also found that none of three allelic *ago2ab* mutants showed defects in growth and development after CMV-∆2b infection (Fig. 4a). After wild-type CMV-Fny infection, these *ago2ab* mutants displayed disease symptoms similar to *ago2a*, *ago2b* single mutants, or wild-type Micro-Tom plants (Fig. 4a, Supplementary Data Fig.S8a). Thus, AGO2a and AGO2b do not function redundantly to regulate disease symptom induction in tomato after viral infection.

We further examined CP protein and viral RNA accumulation in *ago2ab* double mutants using western or northern blot. It was found that CP protein and viral RNAs were significantly increased in these *ago2ab* knockout mutants after infection with either wild-type CMV-Fny or CMV-∆2b, compared with wild-type Micro-Tom plants (Fig. 4b–d, top, Supplementary Data Fig. S8b–g, top). vsiRNA accumulation in these *ago2ab* double mutants was also increased compared with wild-type Micro-Tom plants (Fig. 4b-4d, bottom, Supplementary Data Fig. S8b–g, bottom). To further demonstrate variation in accumulation, we also statistically calculated relative accumulations of CP, viral RNAs and vsiRNAs in the *ago2ab* mutant based on three replicate results for each different *ago2ab* mutant. It was found that, after infection with CMV-Fny or CMV-Δ2b, viral CP was significantly increased by ~115 or ~84% in the *ago2ab* mutant compared with wild-type Micro-Tom (Fig. 4e), and viral RNAs was significantly increased by ~68 or ~68% (Fig. 4f). Further analysis of RT–qPCR targeting viral *RdRp* confirmed the significant elevation of viral accumulation in *ago2ab* mutants (Supplementary Data Fig. S6g–i). However, although vsiRNAs were significantly increased by ~80 or ~87% (Fig. 4g), the relative accumulation of CP, viral RNAs or vsiRNAs in *ago2ab* double mutant was comparable to that in *ago2a* single knockout mutants (Fig. 2e–g). These results indicate that only AGO2a plays important roles in antiviral RNAi, and AGO2a and AGO2b do not function redundantly in antiviral defense in tomato.

### Expression pattern underlying the antiviral function of AGO2a and AGO2b in tomato

**Figure 5:**
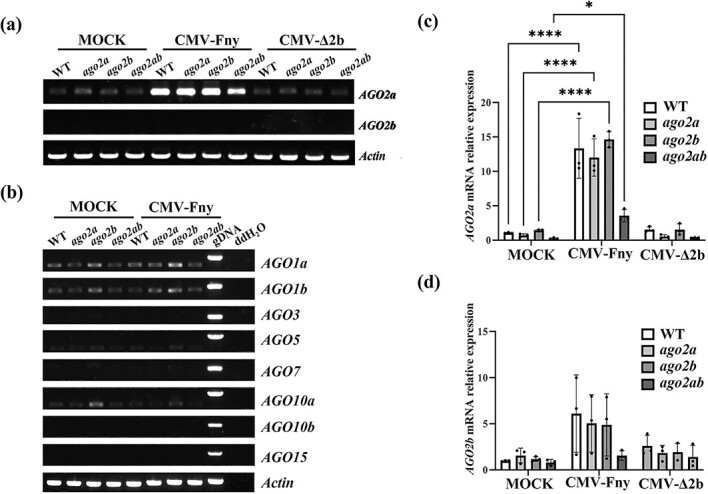
Expression of AGO genes in different genotypes after infection with CMV-Fny or CMV-Δ2b. **a** Semi-quantitative RT–PCR analysis of *AGO2a* and *AGO2b* genes in wild-type (WT), *ago2a*, *ago2b*, and *ago2ab* in Micro-Tom. Experiments were repeated three times with similar results, shown in Supplementary Data Fig. S9a and b. (**b**) Semi-quantitative RT–PCR analysis of AGO genes in WT, *ago2a*, *ago2b*, and *ago2ab* with buffer C (MOCK) or CMV-Fny infection. Experiments were repeated three times with similar results, shown in Supplementary Data Fig. S9d and e. All PCR products were segregated in 1.2% agarose gel after 28 cycles. **c**, **d** Relative *AGO2a* (**c**) and *AGO2b* (**d**) expression in mutant tomato plants inoculated with CMV-Fny or CMV-Δ2b at 19 dpi. Error bars represent the standard deviation. Experiments were repeated three times with similar results. Asterisks indicate a statistically significant difference (^*^P < .05, ^****^P < .0001).

The distinct difference in antiviral defense between AGO2a and AGO2b prompted us to find the underlying mechanism. We examined their transcription level for the two tandem-repeated homologs in tomato before and after viral infection. RT–PCR analysis indicated that *AGO2a* was substantially expressed in tomato plants and its expression was not affected in our *ago2a*, *ago2b*, or *ago2ab* mutants compared with wild-type Micro-Tom plants (Fig. 5a, Supplementary Data Fig.S9a–c). Interestingly, we found that *AGO2a* mRNA expression was drastically elevated after wild-type CMV-Fny infection, though it was not clear after CMV-∆2b infection (Fig. 5a, Supplementary Data Fig.S9a and b). However, *AGO2b* expression could not be detected in these plants before or after infection of either wild-type CMV-Fny or CMV-∆2b by RT–PCR analysis (Fig. 5a, Supplementary Data Fig.S9a–c). Further quantitative RT–PCR analysis demonstrated the same expression pattern of *AGO2a* and *AGO2b* in these plants, except that *AGO2a* and *AGO2b* mRNA expression was modestly decreased in *ago2ab* mutants after CMV-Fny infection (Fig. 5c and d).

We also examined transcriptional expressions of other tomato AGOs. RT–PCR results showed that none of them was induced by infection with CMV-Fny or CMV-∆2b, and their expressions were not affected in *ago2a*, *ago2b*, or *ago2ab* mutants (Fig. 5b, Supplementary Data Fig.S9d and e), indicating that *AGO2a* was the sole effector of antiviral RNAi that would be significantly induced against viral infection in tomato plants.

**Figure 6:**
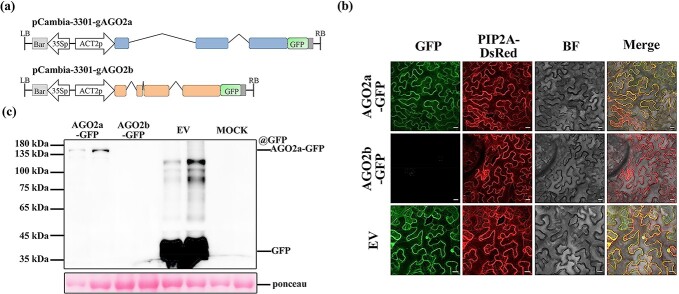
Variation of expression between AGO2a and AGO2b. **a** Schematic diagram to illustrate the vector pCambia-3301-gAGO2a/gAGO2b, used for AGO2a and AGO2b transient expression in the leaves of *N. benthamiana*. **b** Subcellular localization of AGO2a and AGO2b fused to GFP transiently expressed in *N. benthamiana*. Scale bar, 20 μm. **c** Western blot analysis of AGO2a and AGO2b fused to GFP protein transiently expressed in *N. benthamiana* leaves.

We further cloned genomic fragments of the *AGO2a* or *AGO2b* gene into expression vector pCambia-3301 to be fused with GFP in their C-terminals and driven by *ACTIN2* promoter (Fig. 6a), then examined the subcellular localization of both GFP-fused AGO2a and AGO2b proteins. In transient expression, it was found that strong AGO2a-GFP signals were found to co-localize with endomembrane marker PIP2A in the leaves of *N. benthamiana* when observed under a confocal microscope, while GFP signal expressed from empty vector was distributed in different subcellular structures, including the endoplasmic reticulum, cytosol, and nuclei (Fig. 6b). However, AGO2b-GFP signal was barely detected on endo-membrane compared with AGO2a-GFP (Fig. 6b). We further found that AGO2a-GFP protein with correct 130-kDa molecular weight could be readily detected by Western blot, but AGO2b-GFP protein could not be detected in the transient expression (Fig. 6c). Thus, these variations of expression pattern between AGO2a and AGO2b are well consistent with their distinct functions in antiviral RNAi in tomato, and probably underlie their roles in antiviral defense in tomato.

## Discussion

AGO2 is one key effector mediating antiviral RNAi immunity in plants [9, 16, 59, 60]. Compared with the single AGO2 in *Arabidopsis* [61], two differentiated AGO2 homologs, AGO2a and AGO2b, have evolved in tomato [33]. In this research, we successfully generated *ago2a*, *ago2b*, and *ago2ab* knockout mutants using CRISPR-Cas9 (Fig. 1) and infected them with wild-type CMV-Fny or VSR-deficient CMV, CMV-∆2b, to investigate their antiviral function in tomato (Figs 2–4, Supplementary Data Figs S5–S8). It was found that only AGO2a was induced to function in antiviral defense in tomato (Fig. 5, Supplementary Data Fig. S9); AGO2b should be a pseudogene without function due to the diminished expression level of AGO2b in tomato (Fig. 6, Supplementary Data Fig. S9). Thus, for the first time our research revealed the distinct function of two differentiated AGO2 effectors in antiviral immunity in tomato.

Interestingly, we found that AGO2a could defend against infection of not only VSR-deficient CMV but also wild-type CMV-Fny in tomato (Fig. 2, Supplementary Data Figs S5 and S6), which is different from previous findings in *Arabidopsis*, in which wild-type CMV-Fny can be efficiently replicated and propagated in both wild-type *Arabidopsis* plants and antiviral RNAi-defective mutants because VSR 2b almost completely inhibits antiviral RNAi in wild-type *Arabidopsis* plants [26, 56–58, 62]. Therefore, our findings here indicated that antiviral RNAi immunity evolves to protect against infection of wild-type virus with potent VSR in tomato. This function of antiviral RNAi may be shared in varieties of other plants.

In addition, our results showed that wild-type CMV-Fny but not VSR 2b-deficient CMV (CMV-∆2b) caused disease symptoms in tomato plants, indicating the critical roles of 2b in disease symptom induction (Figs 2–4, Supplementary Data Figs S5, S7, andS8). Since wild-type CMV-Fny was abundantly accumulated to a much higher extent in tomato plants than CMV-∆2b, it could be that viral accumulation exceeded a threshold such that plants cannot maintain regular homeostasis for normal plant growth and development. However, it cannot be excluded that 2b may cause plant disease symptoms by directly interfering in growth and developmental processes in tomato [56, 62].

We also found that, just like wild-type Micro-Tom plants, *ago2a*, *ago2b*, and *ago2ab* mutants did not show disease symptoms after CMV-∆2b infection, although virus accumulation was significantly increased in these mutants compared with wild-type Micro-Tom plants (Figs 2–4). After wild-type CMV-Fny infection, *ago2a*, *ago2b*, and *ago2ab* mutants did not show enhanced disease symptoms compared with wild-type Micro-Tom plants either, although viral accumulation was also dramatically increased in these mutants (Fig. 2–4, Supplementary Data Figs S5, S7, andS8). These results suggested that, unlike in *Arabidopsis* [16, 26, 27, 56], AGO2-mediated antiviral RNAi mainly inhibits viral accumulation but does not play a major role in preventing disease induction in tomato.

Therefore, our findings together show that novel mechanisms and specialized function of antiviral RNAi may be developed to counter viral aggression in tomato. It is probable that during the arms race between host and viruses, tomato plants gain new arsenals to attenuate the inhibitory effect of VSR on antiviral RNAi, so that antiviral RNAi in tomato can counteract infection with and propagation of wild-type viruses. However, novel mechanisms in tomato other than antiviral RNAi may play major roles in promoting plant tolerance to maintain health after viral infection. It will be very interesting to find these detailed mechanisms in the future.

## Materials and methods

### Viruses and plant materials

Wild-type virus CMV-Fny is a strain of CMV subgroup 1, isolated and cloned from a muskmelon farm in New York [63]. CMV-Δ2b is a mutant virus of CMV-Fny. In CMV-Δ2b, the third codon UUG in the 2b open reading frame (ORF) encoded by CMV-Fny is mutated to the stop codon UAG, and three AUG codons at the 1st, 8th and 18th positions in 2b ORF are mutated to ACG so that the amino acids encoded by the overlapping part of the 2a ORF are not changed [16].

Tomato cv. Micro-Tom was used as wild-type in this study. *ago2a*, *ago2b*, and *ago2ab* knockout mutants were generated in the Micro-Tom background by CRISPR/Cas9 genome editing.

### Construction of genome editing vector

Two sequences (Solyc02g069260 and Solyc02g069270) of the tomato *AGO2* gene were identified using the Sol genomics network database [64] according to the reported tomato AGO2 sequence [33]. Target sites of these two sequences for editing the tomato AGO2 genome were selected using the online tool CCTop-CRISPR/Cas9 target online predictor and Cas-OFFinder [65, 66]. The pHEE401 vectors are used to generate homozygous mutants for two target genes in *Arabidopsis* with high efficiency [67]. To construct pHEE401-AGO2a-sgRNA, pHEE401-AGO2b-sgRNA, and pHEE401-AGO2ab-sgRNA binary vector, target-specific sgRNA expression cassettes were cloned into the pHEE401 backbone. Briefly, fragments of the sgRNA expression cassettes were amplified from pCBC-DT1T2_tomatoU6 with primer pairs listed in Supplementary Data Table S1, and inserted into pHEE401 vectors using the BsaI restriction enzyme site. Subsequently, these three recombinant vectors were each transformed by heat shock into *Agrobacterium tumefaciens* strain GV3101. Primers used in this study are listed in Supplementary Data Table S1.

### Tomato transformation

*Agrobacterium*-mediated transformations of tomato cotyledons were performed to generate *ago2a*, *ago2b*, and *ago2ab* knockout transgenic tomato plants, as described in previous research [68]. Briefly, cotyledon segments from aseptic seedlings were placed on Murashige and Skoog (MS) medium and precultured in the dark for 2 days. Then, cotyledon explants were soaked in MS liquid medium containing *Agrobacterium* for 10 minutes, and also co-cultivated on MS medium in the dark for 2 days. Next, these cotyledon explants were transferred to a callus induction medium containing 75 mg l^−1^ kanamycin to select transgenic cells. When small shoot buds were induced from callus, they were transferred to shoot elongation medium containing 50 mg l^−1^ kanamycin. Shoots (~1.5 cm tall) then were excised from shoot buds and inserted in rooting medium without hormones for root regeneration. Finally, well-rooted plants were planted in a greenhouse at 26°C with a 16-hours light/8-hours dark photoperiod and light intensity of 20 000 lux.

### DNA extraction and mutant identification

To detect gene editing, genomic DNA was extracted from tomato leaves using a CTAB method as described in previous research [69]. The DNA fragments containing target sites were amplified by PCR with the primer pairs listed in Supplementary Data Table S1. The PCR products were then sequenced by Sanger sequencing to analyze mutations. Positive mutant plants (without Cas9) were planted in the greenhouse at 26°C with a 16-hours light/8-hours dark photoperiod and light intensity of 20 000 lux.

### Virus infection

Viruses were propagated in tobacco (*N. benthamiana*) and purified according to a published protocol [70]. Micro-Tom seeds were germinated and grown in an insect-free growth room at 24°C with a 10-hours light/14-hours dark photoperiod and light intensity of 8000 lux. After 7–9 days, tomato seedlings with two cotyledons were infected with CMV-Fny or CMV-Δ2b. Virus particle solution was diluted with buffer C to the final concentration of 30 ng/μl. Two cotyledons of each seedling were dusted with silicon carbide and mechanically inoculated with Fny-CMV or CMV-Δ2b.

### RNA extraction and northern blot analysis

To analyze viral RNA accumulation, systemically infected leaves were collected at 19 days post-inoculation, and samples were collected from three plants. RNA extraction and northern blots were conducted according to a published protocol [70]. Total or small RNA (10 μg) was loaded onto each lane for northern analysis of viral genomic or vsiRNA accumulation. Northern blots were performed with probes of biotin-dUTP-labeled cDNA or DNA oligonucleotides, as described in previous research [16]. The blot signal was detected with a chemiluminescence image analysis system (Tanon-5200).

### Protein extraction and western blot analysis

Total proteins were extracted from leaf samples according to a method described in published research [71]. Equal amounts of proteins were transferred to PVDF membranes after being separated on 10 or 12.5% SDS–PAGE gels. Viral protein was detected using rabbit polyclonal anti-CP antibody (1:3000; Zoonbio Biotechology) specific to CMV-Fny CP. GFP-fused proteins were detected using rabbit monoclonal anti-GFP antibody (1:3000; Abcam). The blot signal was detected with a chemiluminescence image analysis system (Tanon-5200).

### RT–PCR and quantitative real-time RT–PCR analysis

cDNA was synthesized using the HiScript II cDNA Synthesis Kit (Vazyme) according to the manufacturer’s instructions. AGO genes were subjected to semi-quantitative RT–PCR, and products of 28 cycles were segregated in 1.0% agarose gel. Quantitative real-time RT–PCR (RT–qPCR) was performed using Taq Pro Universal SYBR qPCR Master Mix (Vazyme). The tomato actin gene was used as an internal control and normalizer. Primers used in this study are listed in Supplementary Data Table S1. All experiments were repeated three times.

### Observation of protein subcellular localization

For subcellular localization, tomato *AGO2a* and *AGO2b* genomic DNAs were amplified with primer pairs listed in Supplementary Data Table S1. DNA sequences were integrated into p3301vectors between the SpeI and SmaI sites and each was transformed by heat shock into the *A. tumefaciens* strain GV3101. *A. tumefaciens* cells harboring p3301-EGFP, p3301-EGFP-AGO2a or p3301-EGFP-AGO2b were infiltrated separately with PIP2A-DsRed (membrane marker) into the fifth or sixth true leaves of *N. benthamiana*. The final densities of *A. tumefaciens* cells were equivalent to an A600 of 0.5. Leaves were examined for GFP signal at 36 hours after agroinfiltration by fluorescence microscopy (Leica DMI 6000B with an L5 filter block containing a 480/40-nm excitation filter, a 505 nm dichroic mirror, and a 527/30-nm barrier filter for GFP fluorescence), and images were taken using Leica LAS AF software.

## Acknowledgements

The authors thank professor Chuanyou Li for sharing the construct pHEE401, and Chuanlong Sun and Yuzhen Mei for experimental advice; and all members of Zhongxin Guo’s and Yanhong Han’s groups for helpful suggestions. This work was supported by the National Natural Science Foundation of China (31870146 and 32160619), the Science Foundation of Fujian province (2020 J02014), ‘Hundred Talent’ of Fujian Province, Yunnan Seed Industry Joint Laboratory (202205AR070001), and the Science and Technology Major Project of Yunnan (202202AE090022).

## Author contributions

Z.X.G. and L.L.Z. designed the experiments, analyzed the data, and wrote the manuscript. Z.Z.K provided advice and support for the research. L.L.Z., Y.F.C., X.M.X., H.Y.G., and J.M.C. performed the experiments. All authors read and approved the manuscript.

## Data availability

All data needed to evaluate the conclusions in the paper are present in the paper and/or the supplementary materials. All newly generated plant or vector materials are available for sharing upon request. Sequence data used in this study were downloaded from Sol Genomics (Sol Genomics Network). The accession numbers are as follows: *AGO2a* (Solyc02g069260), *AGO2b* (Solyc02g069270), *AGO1a* (Solyc06g072300), *AGO1b* (Solyc03g098280), *AGO3* (Solyc02g069280), *AGO5* (Solyc06g074730), *AGO*6 (Solyc07g049500), *AGO10*a (Solyc09g082830), *AGO10b* (Solyc12g006790), *AGO15* (Solyc03g111760), *Actin* (Solyc11g005330), *NtAGO2* (XM_016629769), *AtAGO2* (AT1G31280), and *OsAGO*2 (XP_015636011).

## Conflict of interest

None declared.

## Supplementary data

Supplementary data is available at *Horticulture Research* online.

## Supplementary Material

Web_Material_uhad043
