# Supplementary material for: AGO2a but not AGO2b mediates antiviral defense against infection of wild-type cucumber mosaic virus in tomato
Source: Hortic Res. 2023 Mar 13;10(5):uhad043. doi: 10.1093/hr/uhad043 (PMC10177002; doi:10.1093/hr/uhad043)
Supplement: Web_Material_uhad043 [file web_material_uhad043.zip › Supplementary Information-revised.docx]

**Supplementary Information**

**AGO2a but not AGO2b mediates antiviral defense against the infection of wildtype Cucumber mosaic virus in tomato**

**Running title: AGO2a protects tomato from the CMV infection**

Liling Zhao^# , 1, 2^, Yingfang Chen^#, 1^, Xingming Xiao^1^, Haiying Gao^1^, Jiamin Cao^1^, Zhongkai Zhang*^2^, Zhongxin Guo*^1^

1, Vector-borne Virus Research Center, State Key Laboratory for Ecological Pest Control of Fujian and Taiwan Crops, College of Plant Protection, Fujian Agriculture and Forestry University, Fuzhou 350002 China.

2, Key Lab of Agricultural Biotechnology of Yunnan Province, Biotechnology and Germplasm Resources Research Institute, Yunnan Academy of Agricultural Sciences, Kunming, China.

**
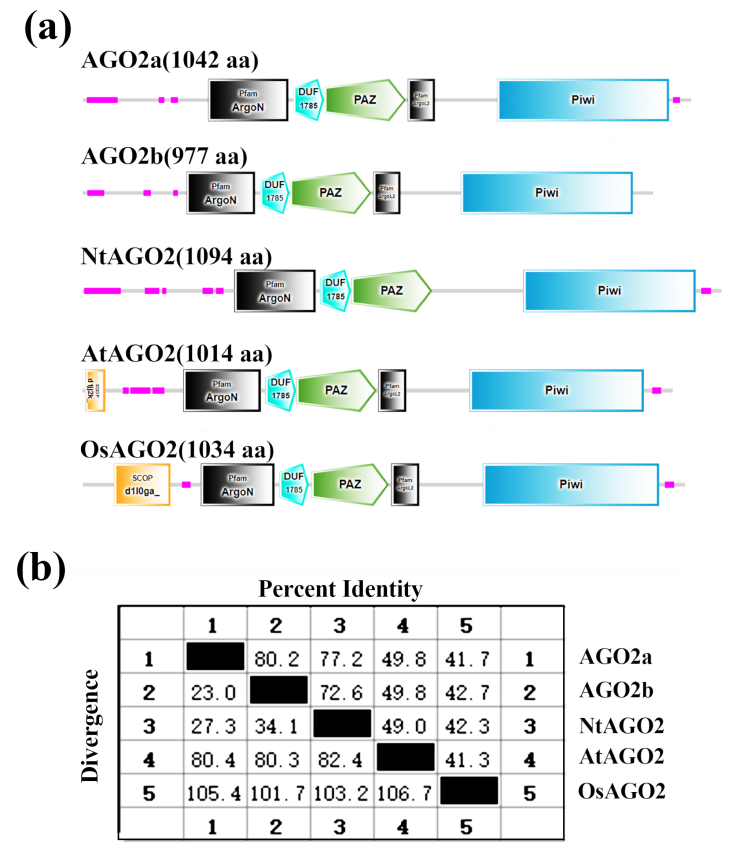
**

**Figure S1.** Protein structures and similarity of amino acid sequences of AGO2 proteins. (**a**) Schematic diagrams were illustrated protein structures of the AGO2, originated from tomato, tobacco, Arabidopsis and rice. Protein structures of AGO2 were produced using the online tool SMART (<http://smart>.embl.de). (**b**) Similarity of amino acid sequences of AGO2a with other AGO2s. Multiple sequence alignments were performed using MegAlign suite included in DNASTAR software version 11.

**
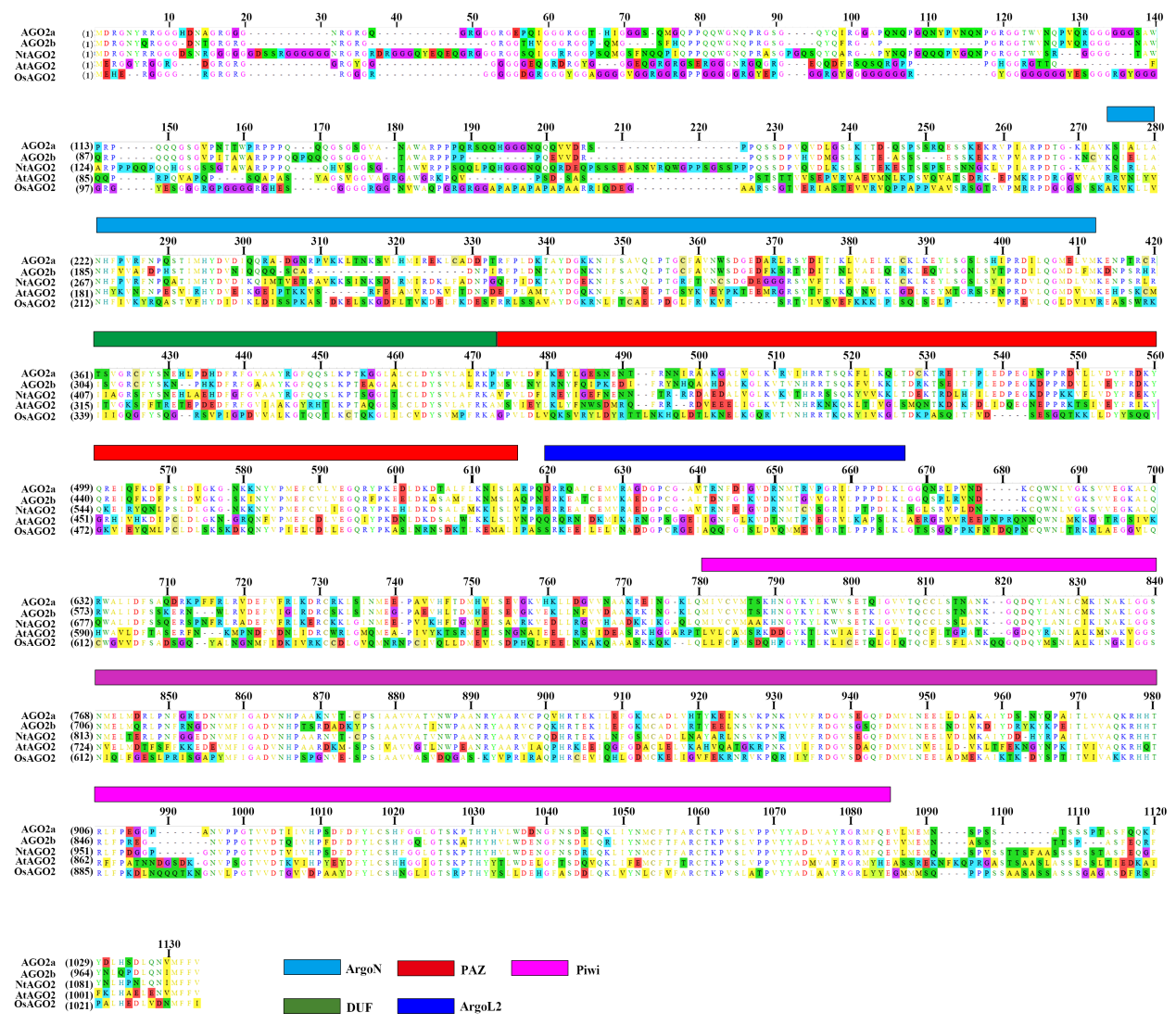
**

**Figure S2.** Multiple alignments of AGO2 proteins originated from tomato, tobacco, Arabidopsis and rice. The major functional domains of AGO2 are indicated by colored boxes.

**
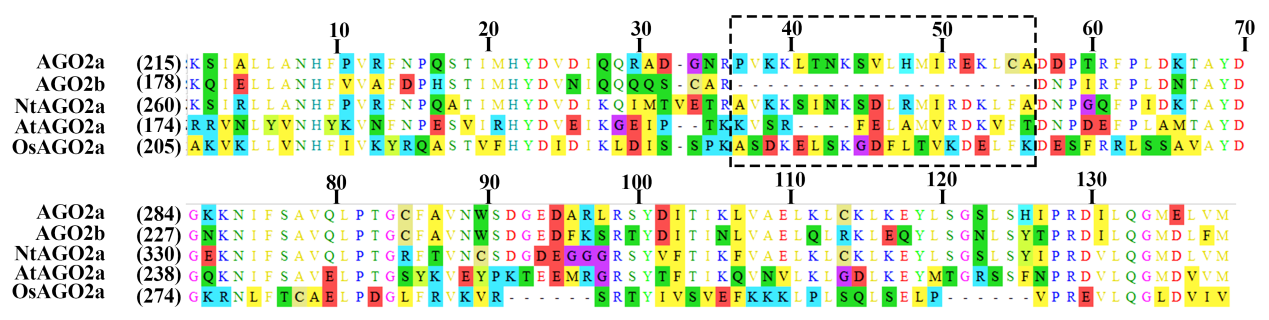
**

**Figure S3.** Multiple alignment of ArgoN domain of AGO2a proteins. Rectangular box indicates the deleted region only in AGO2b, but retained in AGO2a, NtAGO2, AtAGO2 and OsAGO2.


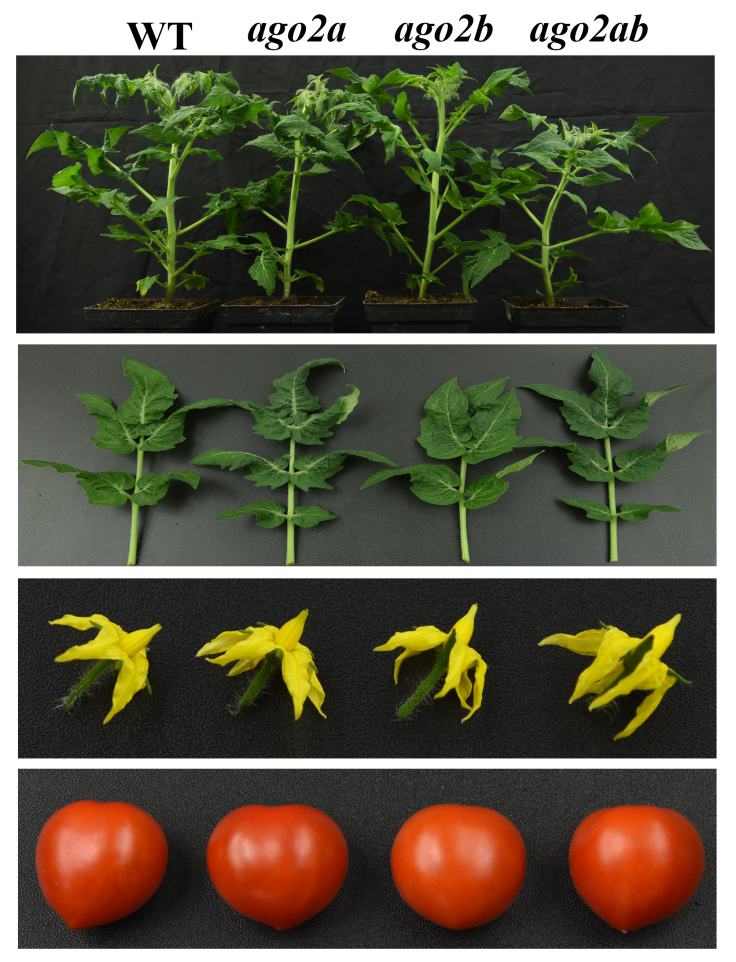


**Figure S4.** *ago2a*, *ago2b* and *ago2ab* do not show defects in growth and development compared to WT.


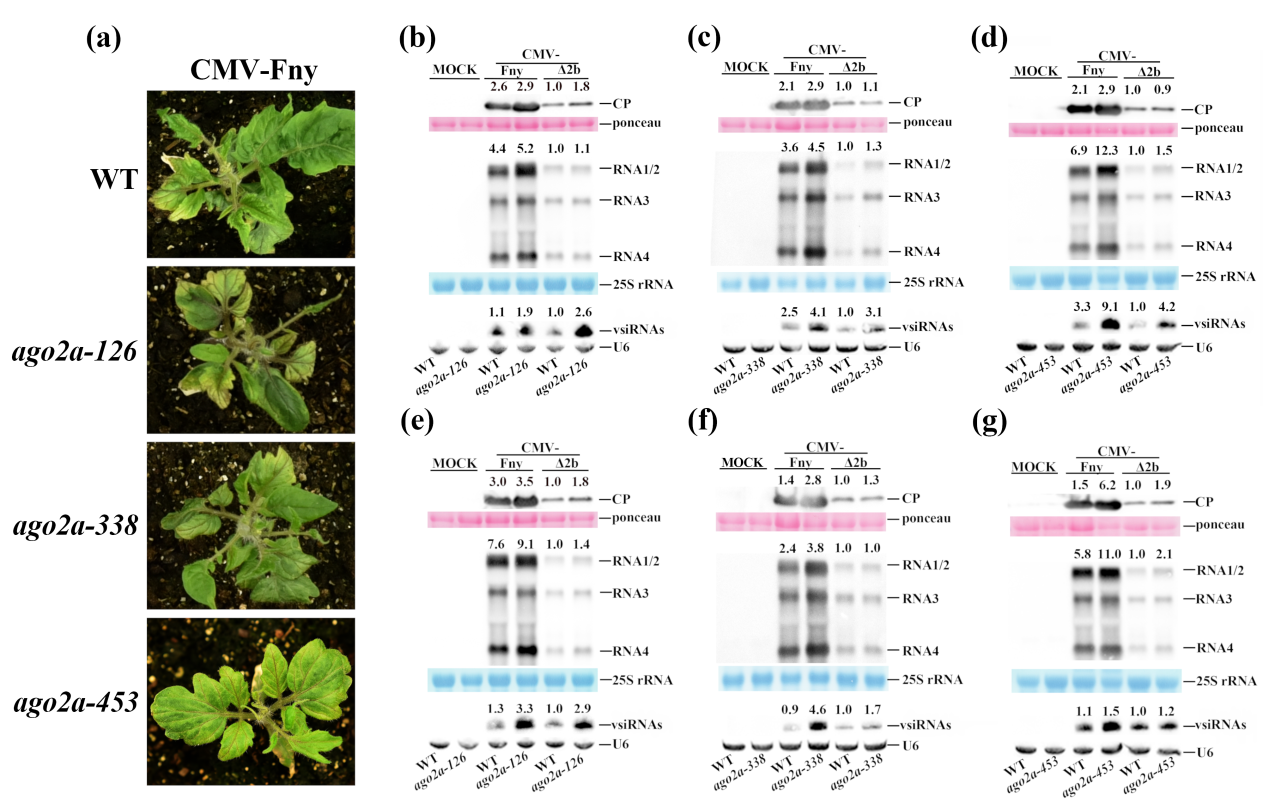


**Figure S5.** *ago2a* mutants are defective in antiviral immunity. (**a**) WT and three lines of *ago2a* mutants (*126*, *338* and *453*) were photographed 19 dpi with CMV-Fny. Accumulation of viral protein, RNAs and vsiRNAs in WT and mutant lines of *ago2a-126* (**b**, **e**), *ago2a-338* (**c**, **f**) and *ago2a-453* (**d**, **g**). Blot-experiments per mutant line were repeated three times. The figure above shows the results of two additional experimental replicates. Ponceau, 25S rRNA and U6 RNA were used as the loading controls. Values at the top panel represent corresponding hybridization signal intensity of viral CP, RNAs 1-4, and vsiRNAs. The intensity of WT inoculated with CMV-∆2b was set as 1.


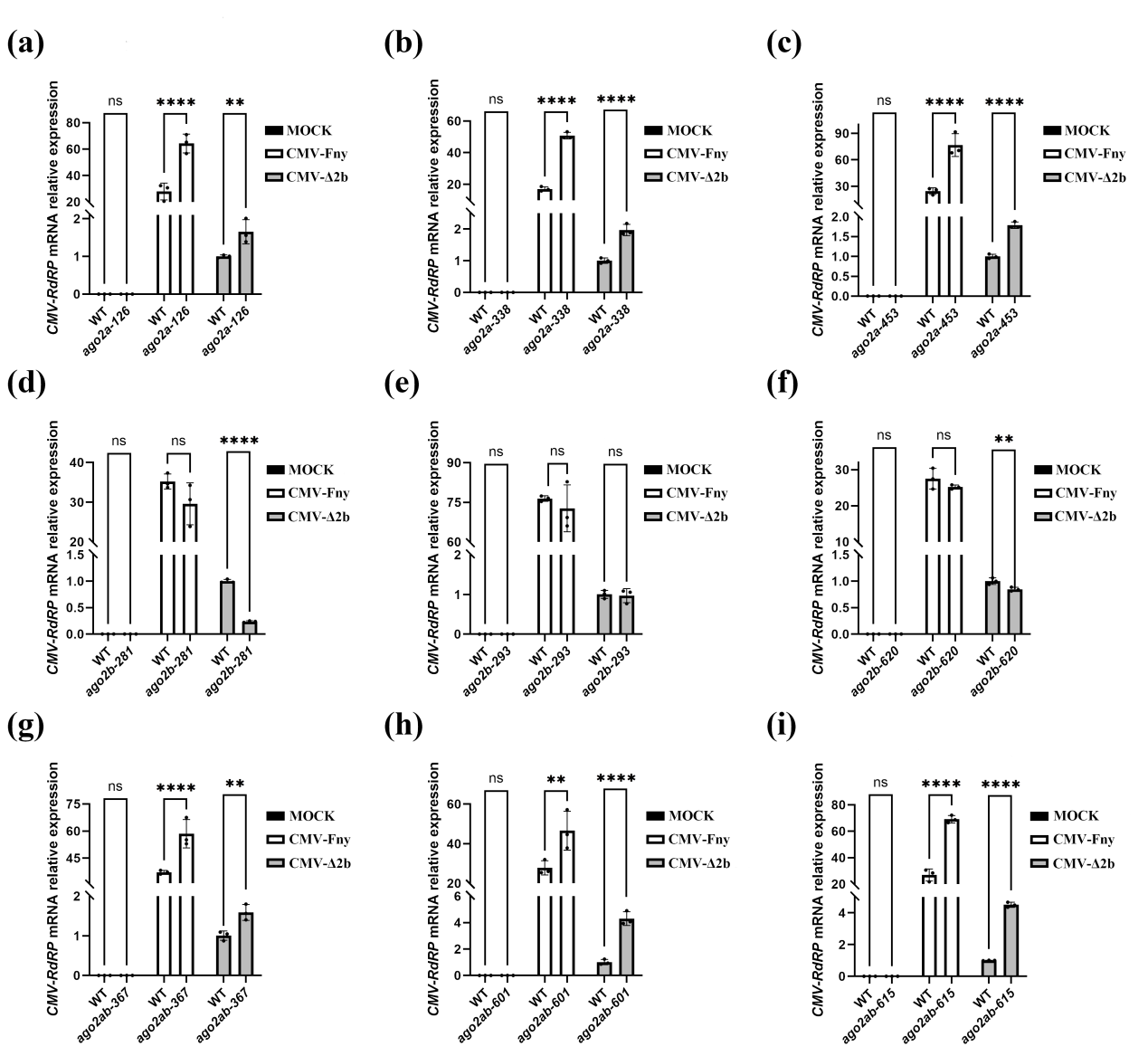


**Figure S6.** Relative CMV-RdRp mRNA accumulation in WT or mutant tomato plants inoculated with CMV-Fny or CMV-Δ2b at 19 dpi. Relative CMV-RdRp mRNA accumulation in WT and mutant lines of *ago2a-126* (**a**), *ago2a-338* (**b**), *ago2a-453* (**c**), *ago2b-281* (**c**), *ago2b-293* (**d**), *ago2b-620 (***e***)*, *ago2ab-367* (**f**), *ago2ab-601* (**g**) and *ago2ab-615* (**h**). Error bars represent SD. Experiments were repeated three times with similar results. Asterisks indicate a statistically significant difference (***p*<0.01, *****p*<0.0001).


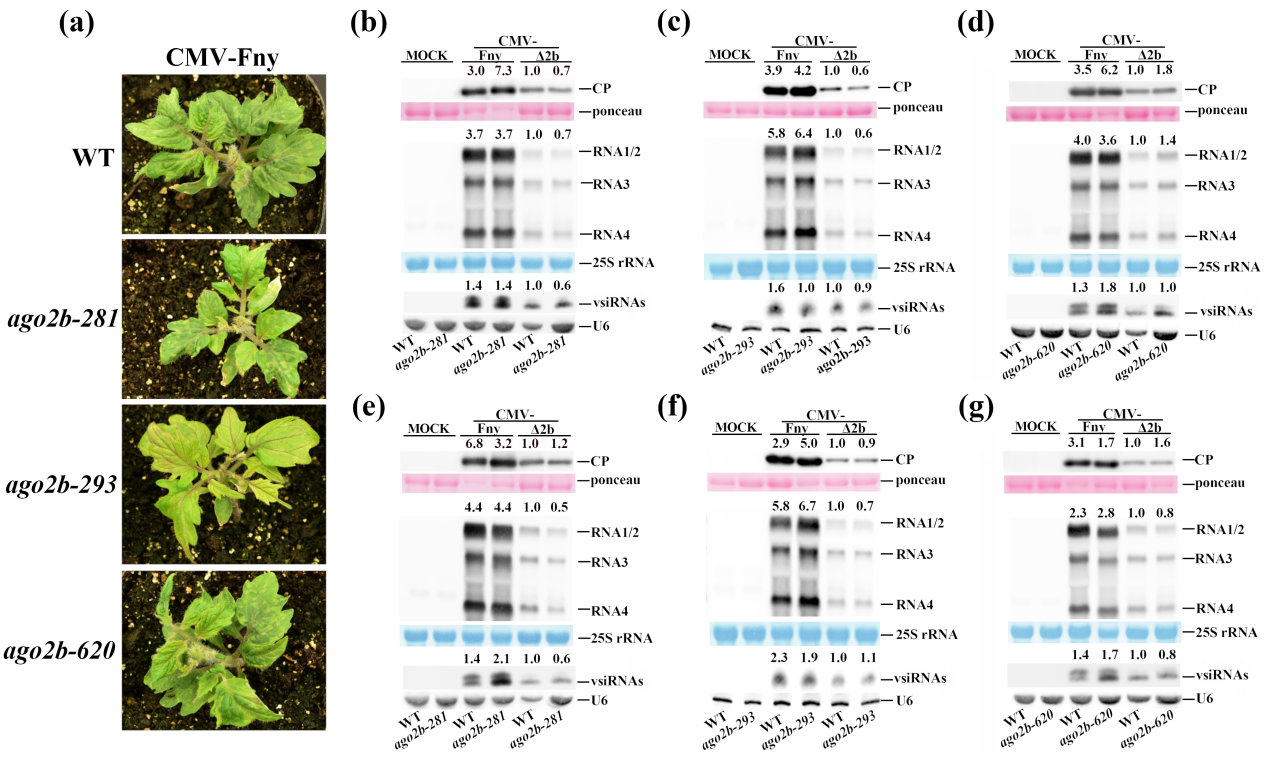


**Figure S7.** AGO2b does not function in antiviral immunity in tomato. (**a**) WT and three lines of *ago2b* mutants (*281*, *293* and *620*) were photographed 19 dpi with CMV-Fny. Accumulation of viral protein, RNAs and vsiRNAs in WT and mutant lines of *ago2b-281* (**b**, **e**), *ago2b-293* (**c**, **f**) and *ago2b-620* (**d**, **g**). Blot-experiments per mutant line were repeated three times. The figure above shows the results of two additional experimental replicates. Ponceau, 25S rRNA and U6 RNA were used as the loading controls. Values at the top panel represent corresponding hybridization signal intensity of viral CP, RNAs 1-4, and vsiRNAs. The intensity of WT inoculated with CMV-∆2b was set as 1.


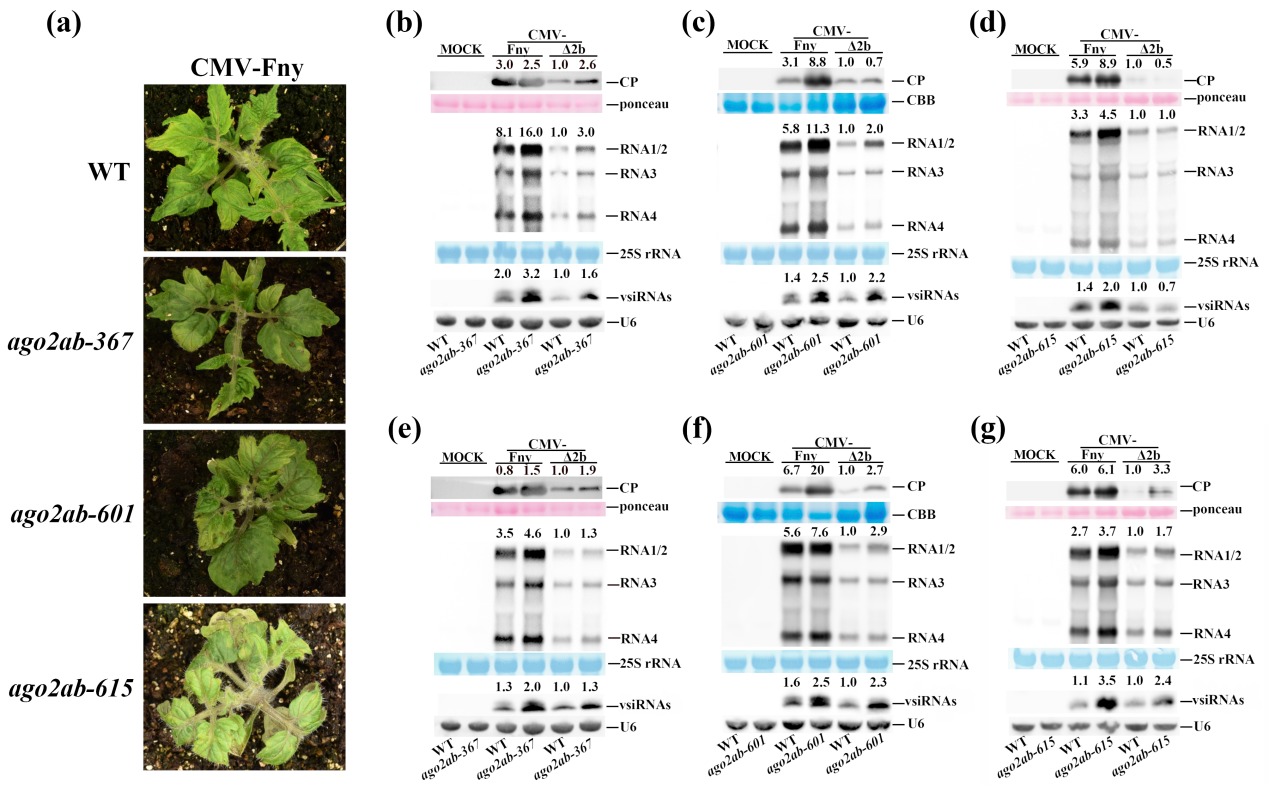


**Figure S8.** AGO2a and AGO2b do not function redundantly in antiviral defense in tomato. (**a**) WT and three lines of *ago2ab* mutants (*367*, *601* and *615*) were photographed 19 dpi with CMV-Fny. Accumulation of viral protein, RNAs and vsiRNAs in WT and mutant lines of *ago2ab-367* (**b**, **e**), *ago2b-601* (**c**, **f**) and *ago2ab-615* (**d**, **g**). Blot-experiments per mutant line were repeated three times. The figure above shows the results of two additional experimental replicates. Ponceau, CBB, 25S rRNA and U6 RNA were used as the loading controls. Values at the top panel represent corresponding hybridization signal intensity of viral CP, RNAs 1-4, and vsiRNAs. The intensity of WT inoculated with CMV-∆2b was set as 1.


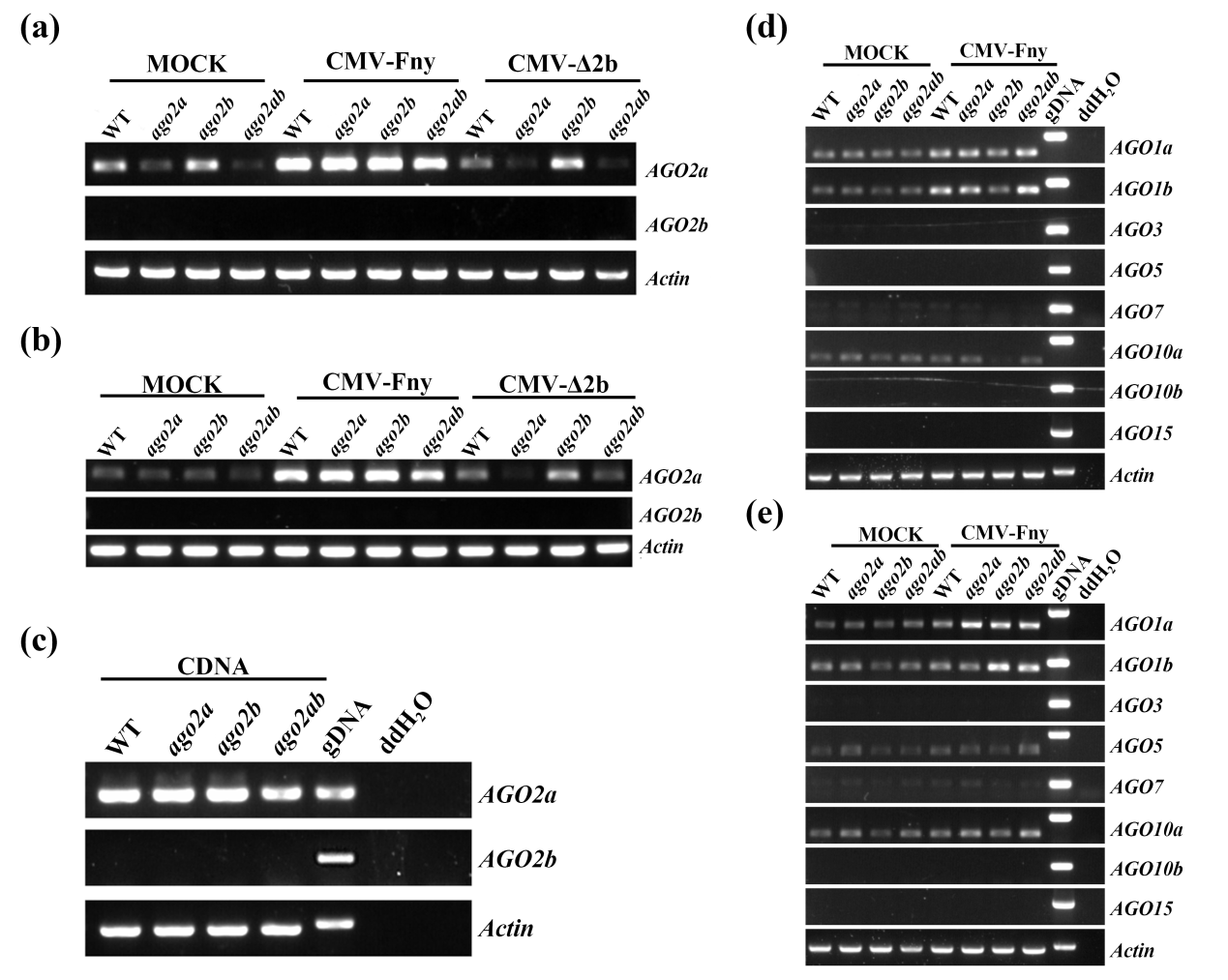


**Figure S9.** Expression of AGO genes in different genotypes inoculated with CMV-Fny or CMV-Δ2b. (**a**, **b**) Semi-quantitative RT-PCR analysis of AGO2a and AGO2b genes in WT, ago2a, ago2b and ago2ab in Micro-Tom. The figure above shows the results of two additional experimental replicates. (**c**) PCR detection of AGO2b and AGO2b genes in Micro-Tom cDNA and genomic DNA (gDNA). PCR products of AGO2b were obtained from gDNA, suggesting that lack of PCR products from cDNA is due to lower gene expression instead of failure of PCR. (**d**, **e**) Semi-quantitative RT-PCR analysis of AGO genes in WT, *ago2a*, *ago2b* and *ago2ab* with buffer C or CMV-Fny infection. The figure above shows the results of two additional experimental replicates.

# Table S1. Primers used in this study

| **Function** | **Primer name** | **Sequence 5' to 3'** | **Reference** |  |
| --- | --- | --- | --- | --- |
| construction of pHEE401 binary vector, used to knockout *AGO2a* gene | *AGO2a*-DT1-BsF | ATATATGGTCTCGATTGATCGTGGGAACTACCGACGGTT | In this study |  |
|  | *AGO2a*-DT1-F0 | TGATCGTGGGAACTACCGACGGTTTTAGAGCTAGAAATAGC | In this study |  |
|  | *AGO2a*-DT2-R0 | AACCCACGGCCTTGTCCTCGACCAATCTCTTAGTCGACTCTAC | In this study |  |
|  | *AGO2a*-DT2-BsR | ATTATTGGTCTCGAAACCCACGGCCTTGTCCTCGACCAA | In this study |  |
| construction of pHEE401 binary vector, used to knockout *AGO2b* gene | *AGO2b*-DT1-BsF | ATATATGGTCTCGATTGGTGGAGCTCCGCCGGGTCGGTT | In this study |  |
|  | *AGO2b*-DT1-F0 | TGGTGGAGCTCCGCCGGGTCGGTTTTAGAGCTAGAAATAGC | In this study |  |
|  | *AGO2b*-DT2-R0 | AACTTGCCAAGCGTTACCACCACAATCTCTTAGTCGACTCTAC | In this study |  |
|  | *AGO2b*-DT2-BsR | ATTATTGGTCTCGAAACTTGCCAAGCGTTACCACCACAA | In this study |  |
| construction of pHEE401 binary vector, used to knockout *AGO2a* and *AGO2b* gene | *AGO2a-*DT1-BsF | ATATATGGTCTCGATTGGTCTGACGGGGAAGATGCTGTT | In this study |  |
|  | *AGO2a*-DT1-F0 | TGGTCTGACGGGGAAGATGCTGTTTTAGAGCTAGAAATAGC | In this study |  |
|  | *AGO2b*-DT2-R0 | AACCGTTGGTAGTTTCCACGGTCAATCTCTTAGTCGACTCTAC | In this study |  |
|  | *AGO2b*-DT2-BsR | ATTATTGGTCTCGAAACCGTTGGTAGTTTCCACGGTCAA | In this study |  |
| Genotyping for *ago2a* mutants | *AG02a*-detect-F | CGAGTTGTTTACTCCTAAG | In this study |  |
|  | *AG02a*-detect-R | CGTTGAACCGGTTGGTTTAC | In this study |  |
| Genotyping for *ago2b* mutants | *AG02b*-detect-F | GACCGTGGAAACTACCAACG | In this study |  |
|  | *AG02b*-detect-R | CCAGAAGATTGAGGACGATC | In this study |  |
| Genotyping for *ago2ab* mutants | *AG02ab*-2a-detect-F | GATCAGAGTCCATCATCTCG | In this study |  |
|  | *AG02ab*-2a-detect-R | GACCTACAGAAGTCCTGCAC | In this study |  |
|  | *AG02ab*-2b-detect-F | GAGTCAGAAGTGTATACAAC | In this study |  |
|  | *AG02ab*-2b-detect-R | CAAGCAGTGATTGGAACACC | In this study |  |
| using for semi-quantitative RT-PCR analysis of *AGO2a* | *AGO2a*-F | TTGGCGAAGGCTATATACGACAG | Miao et al., 2012 |  |
|  | *AGO2a*-R | AGTTGCAGAGCTAGGAGAGTTCATC | Miao et al., 2012 |  |
| using for semi-quantitative RT-PCR analysis of *AGO2b* | *AGO2b*-F | ATCGTTACAAGTATAAACCTGAAATCAC | Miao et al., 2012 |  |
|  | *AGO2b*-R | GGCAGGTGAAGTTGTAGAGCTAGAA | Miao et al., 2012 |  |
| using for semi-quantitative RT-PCR analysis of *AGO1a* | *AGO1a*-F | CTATCAGCCCCCAGTTACGTTTG | Miao et al., 2012 |  |
|  | *AGO1a*-R | ATCACCCTTTTGACATTCTCCTTG | Miao et al., 2012 |  |
| using for semi-quantitative RT-PCR analysis of *AGO1b* | *AGO1b*-F | CAAGACACTGTTGCACATGGGTT | Miao et al., 2012 |  |
|  | *AGO1b*-R | CAAAAATGATTGTAGGACGGTCG | Miao et al., 2012 |  |
| using for semi-quantitative RT-PCR analysis of *AGO3* | *AGO3*-F | CACAGAAATCCAGGACTGAAAAGA | Miao et al., 2012 |  |
|  | *AGO3*-R | CGTTGTAAATCGACAGATATGAAGC | Miao et al., 2012 |  |
| using for semi-quantitative RT-PCR analysis of *AGO5* | *AGO5*-F | TAGTAAGAACATGCCTTTCCTCACC | Miao et al., 2012 |  |
|  | *AGO5*-R | GCAGCTCTTACATCCACGTCATTC | Miao et al., 2012 |  |
| using for semi-quantitative RT-PCR analysis of *AGO7* | *AGO7*-F | GGCTGGCATGTTCGAGATTTC | Miao et al., 2012 |  |
|  | *AGO7*-R | GTGGCACCAGTGAAATAGGCTTG | Miao et al., 2012 |  |
| using for semi-quantitative RT-PCR analysis of *AGO10a* | *AGO10a*-F | CGAGTTAGATGCAATTAGGAAGGC | Miao et al., 2012 |  |
|  | *AGO10a*-R | AATTAGTTTCAGGCATGTCTGGTTC | Miao et al., 2012 |  |
| using for semi-quantitative RT-PCR analysis of *AGO10b* | *AGO10b*-F | CCAGGAGGTGTATGGATACACC | Miao et al., 2012 |  |
|  | *AGO10b*-R | CTCGAGCTTCCACTGATGCAAGC | Miao et al., 2012 |  |
| using for semi-quantitative RT-PCR analysis of *AGO15* | *AGO15*-F | TCTCTTTAGACCAGTTTCAGATAGGG | Miao et al., 2012 |  |
|  | *AGO15*-R | CATCTGAGCTGAAACCAAACGAG | Miao et al., 2012 |  |
| using for RT-qPCR analysis of *AGO2a* | qPCR-*AGO2a*-F | TACCTTCAGGAACAACATTCGA | Miao et al., 2012 |  |
|  | qPCR-*AGO2a*-R | GGGATCTTCAAGGGGAAAAGTA | Miao et al., 2012 |  |
| using for qRT-PCR analysis of *AGO2b* | qPCR-*AGO2b*-F | GTCACGCACTTATGACATTACC | Miao et al., 2012 |  |
|  | qPCR-*AGO2b*-R | CTTGCAGTATATCACGAGGAGT | Miao et al., 2012 |  |
| using for semi-quantitative RT-PCR and RT-qPCR analysis of *ACTIN* | qPCR-actin-F | GGTCCTCTTCCAGCCATCC | Miao et al., 2012 |  |
|  | qPCR-actin-R | CCACTGAGCACAATGTTACCG | Miao et al., 2012 |  |
| using for RT-qPCR analysis of CMV-RdRp accumulation | CMV-RdRp-F | GGATCATGTTAGACTTCGAC | In this study |  |
|  | CMV-RdRp-R | CTCTGGAACGTCAGCATTAC | In this study |  |
| construction of p3301 binary vector, used to observe *AGO2a* subcellular localization | p3301-*AGO2a*-GFP-F | ATCGTGTGTGACCTCGAGACTAGTATGGATCGTGGGAACTACCGAC | In this study |  |
|  | p3301-*AGO2a*-GFP-R | ACCAGGTGGAGGTCCCCCGGGGACGAAAAACATTACGTTCTGC | In this study |  |
| construction of p3301 binary vector, used to observe *AGO2b* subcellular localization | p3301-*AGO2b*-GFP-F | ATCGTGTGTGACCTCGAGACTAGTATGGACCGTGGAAACTACCAAC | In this study |  |
|  | p3301-*AGO2b*-GFP-R | ACCAGGTGGAGGTCCCCCGGGGACAAAAAACATAATGTTCTGC | In this study |  |
