# Supplementary figures and images for: AGO2a but not AGO2b mediates antiviral defense against infection of wild-type cucumber mosaic virus in tomato
Source: Hortic Res. 2023 Mar 13;10(5):uhad043. doi: 10.1093/hr/uhad043 (PMC10177002; doi:10.1093/hr/uhad043)

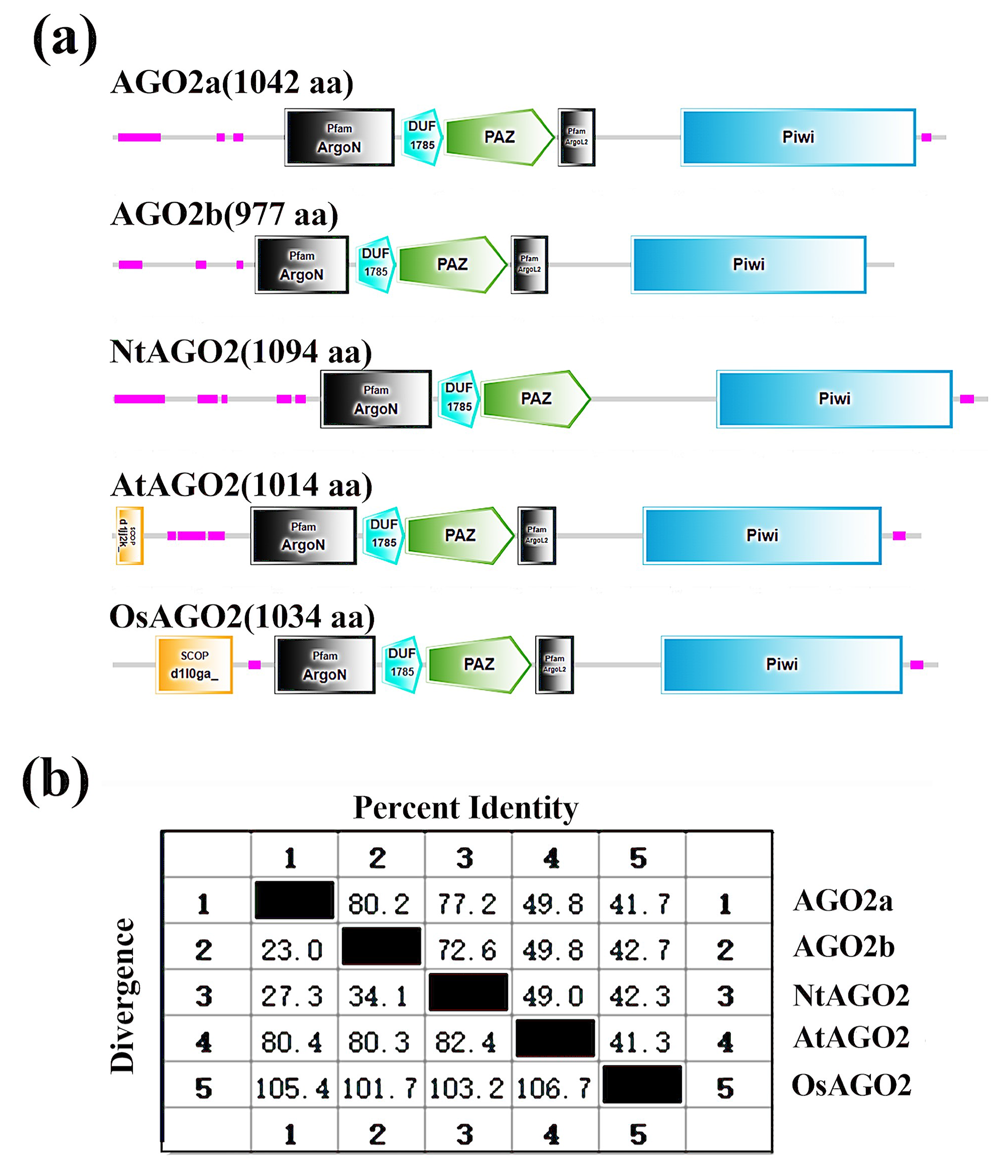

Supplement: Web_Material_uhad043 [file web_material_uhad043.zip › SFig 1.tif]

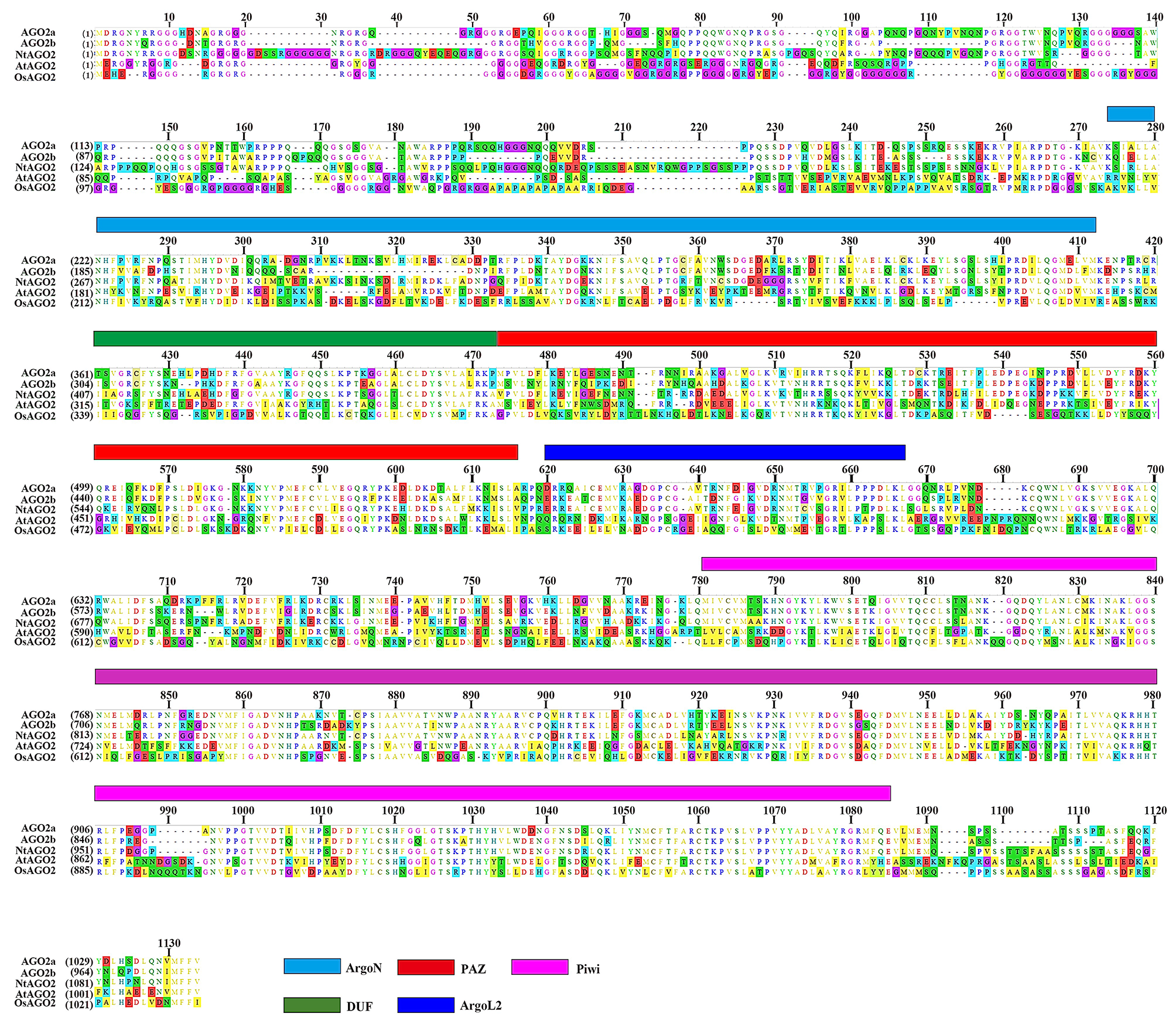

Supplement: Web_Material_uhad043 [file web_material_uhad043.zip › SFig 2.tif]

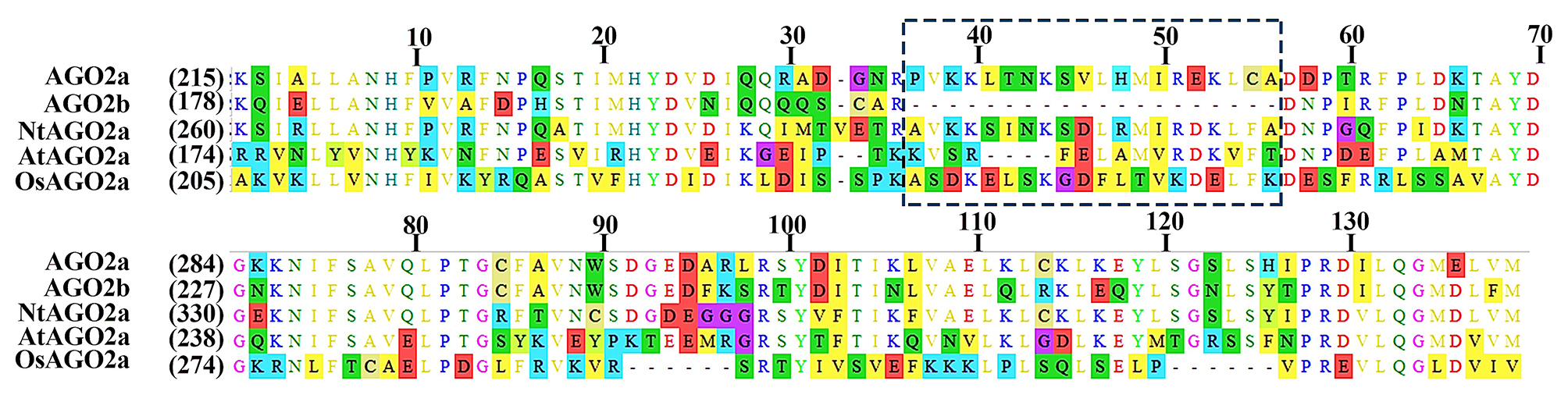

Supplement: Web_Material_uhad043 [file web_material_uhad043.zip › SFig 3.tif]

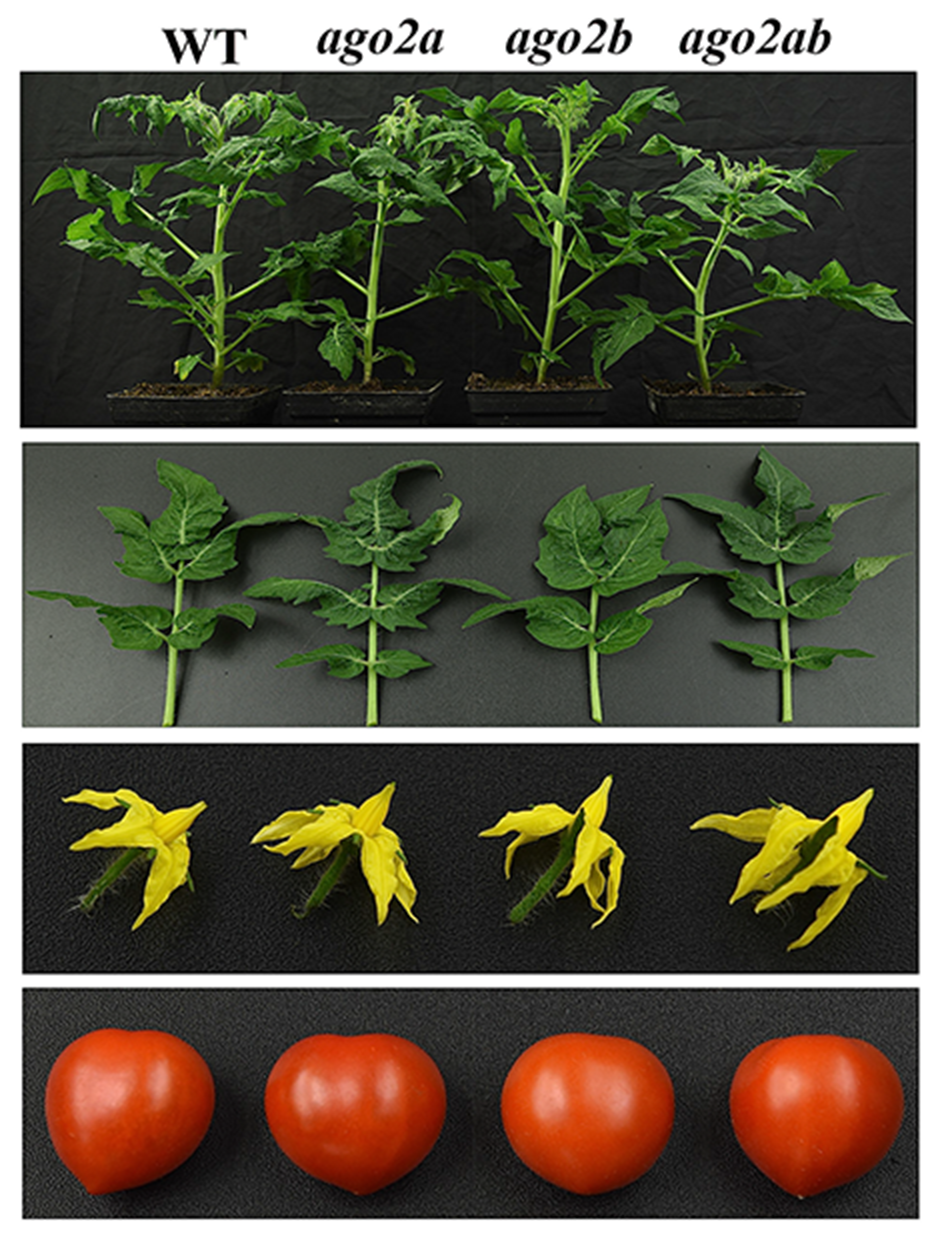

Supplement: Web_Material_uhad043 [file web_material_uhad043.zip › SFig 4.tif]

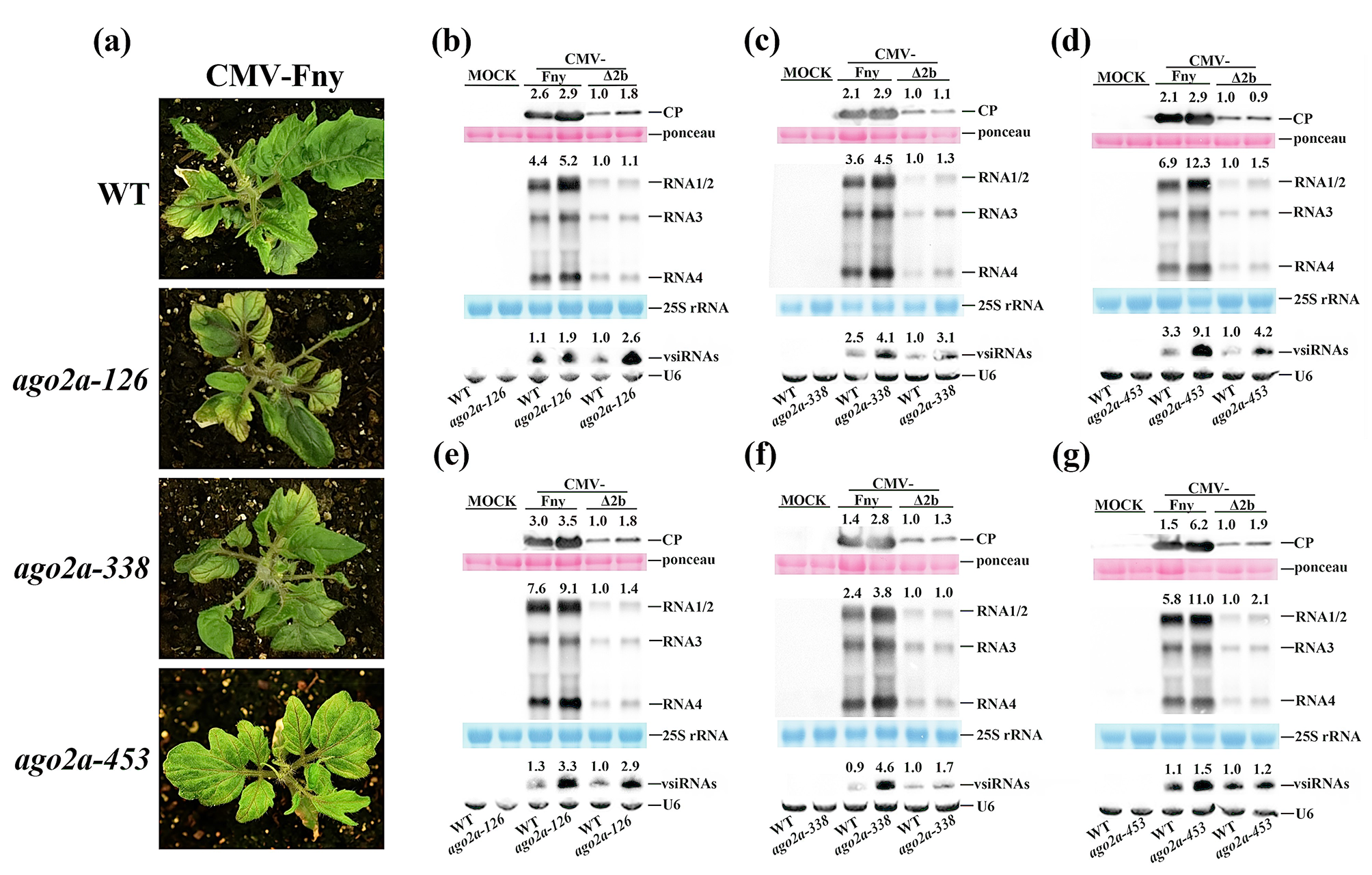

Supplement: Web_Material_uhad043 [file web_material_uhad043.zip › SFig 5.tif]

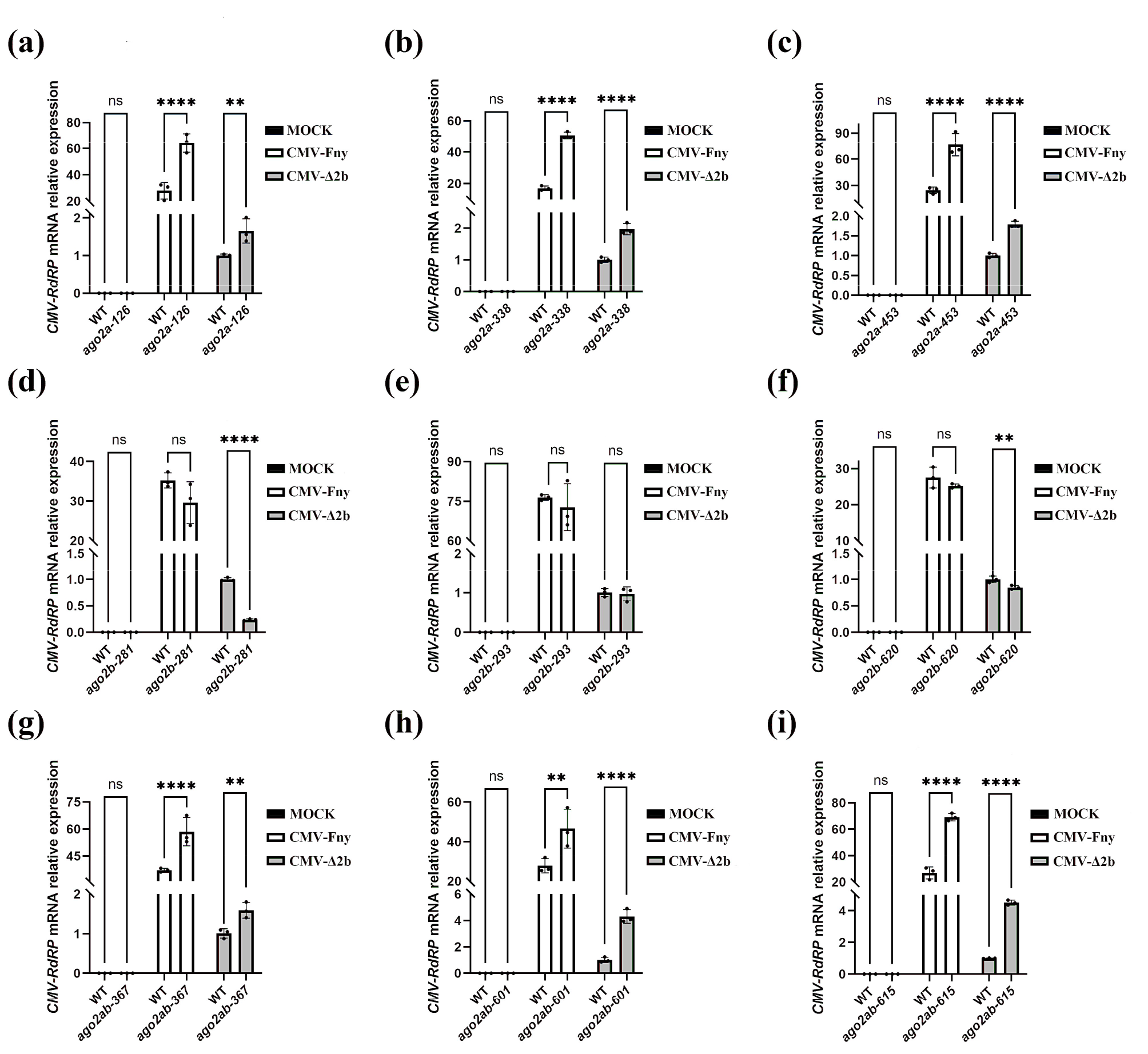

Supplement: Web_Material_uhad043 [file web_material_uhad043.zip › SFig 6.tif]

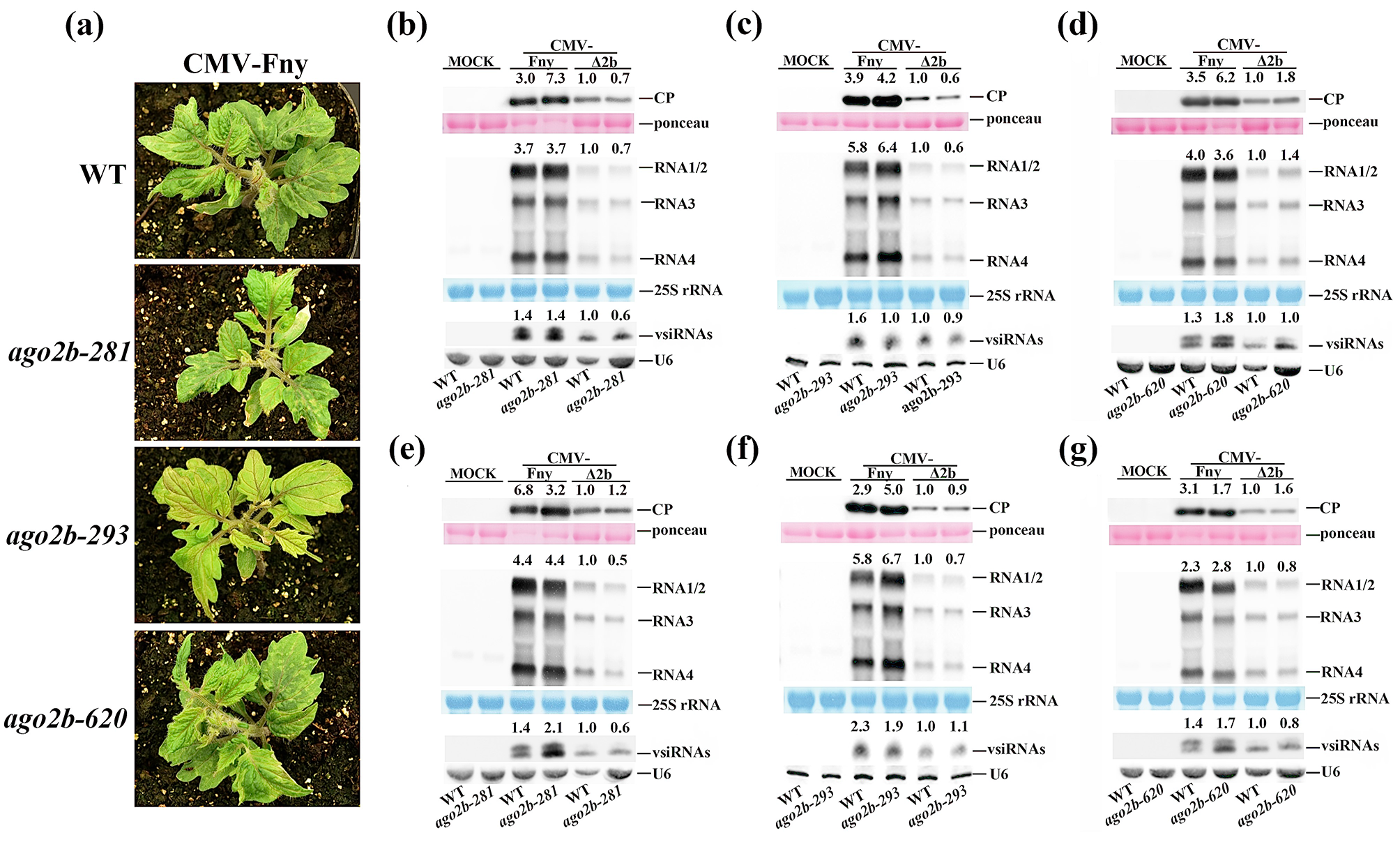

Supplement: Web_Material_uhad043 [file web_material_uhad043.zip › SFig 7.tif]

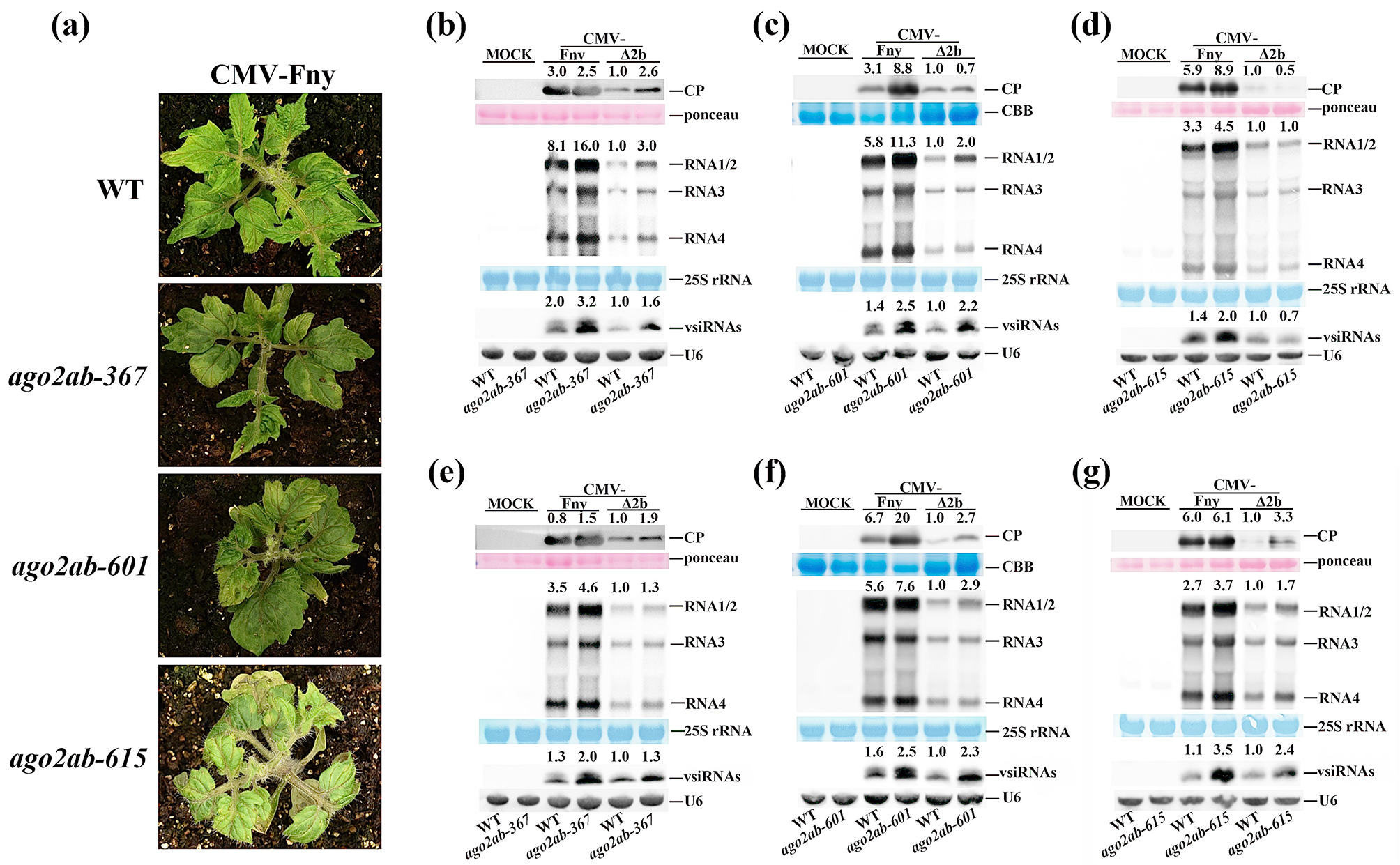

Supplement: Web_Material_uhad043 [file web_material_uhad043.zip › SFig 8.tif]

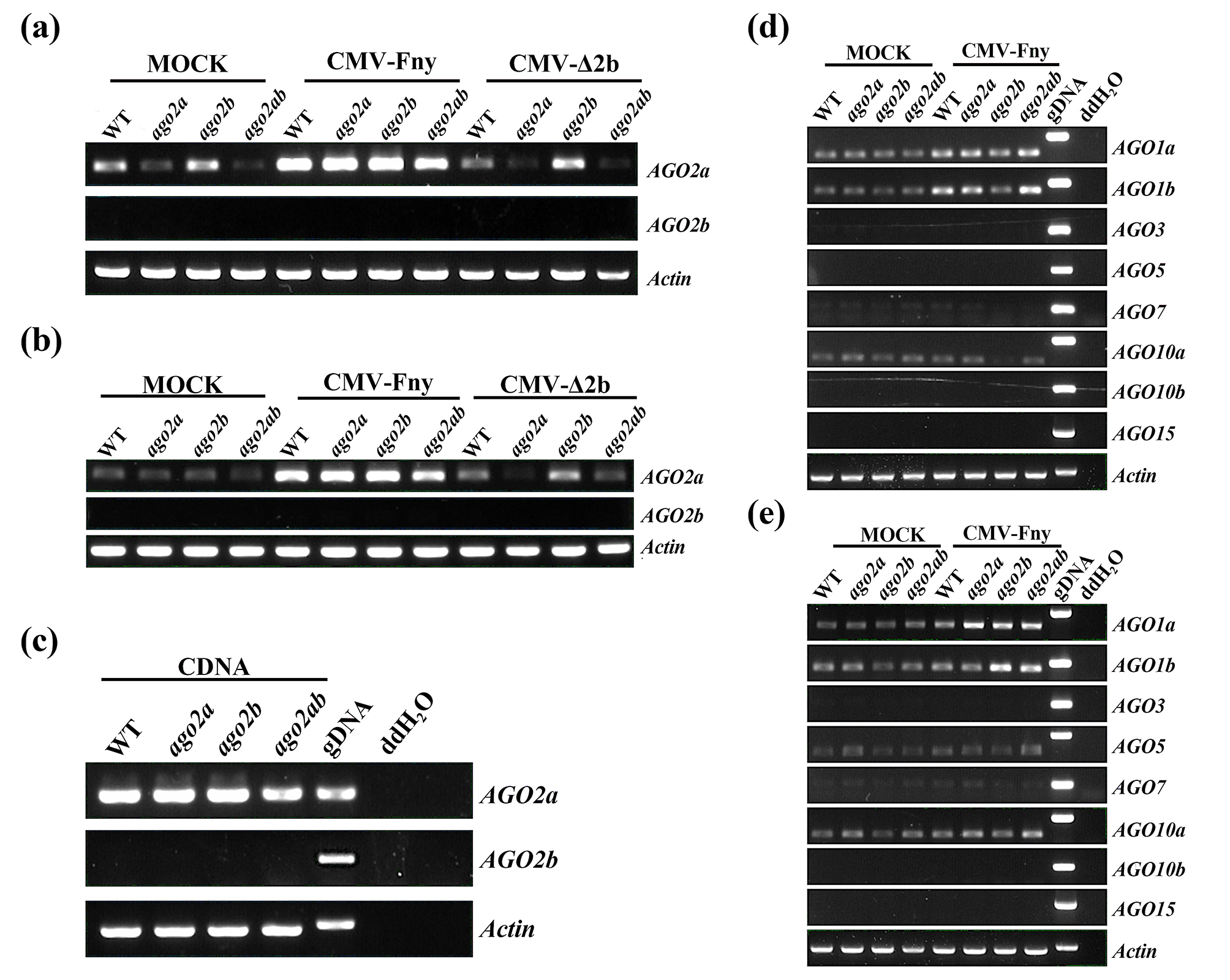

Supplement: Web_Material_uhad043 [file web_material_uhad043.zip › SFig 9.tif]
